# A New Method for Identifying Essential Proteins Based on Network Topology Properties and Protein Complexes

**DOI:** 10.1371/journal.pone.0161042

**Published:** 2016-08-16

**Authors:** Chao Qin, Yongqi Sun, Yadong Dong

**Affiliations:** Beijing Key Lab of Traffic Data Analysis and Mining, Beijing Jiaotong University, Beijing, China; Kings College London, UNITED KINGDOM

## Abstract

Essential proteins are indispensable to the viability and reproduction of an organism. The identification of essential proteins is necessary not only for understanding the molecular mechanisms of cellular life but also for disease diagnosis, medical treatments and drug design. Many computational methods have been proposed for discovering essential proteins, but the precision of the prediction of essential proteins remains to be improved. In this paper, we propose a new method, LBCC, which is based on the combination of local density, betweenness centrality (BC) and in-degree centrality of complex (IDC). First, we introduce the common centrality measures; second, we propose the densities *Den*_1_(*v*) and *Den*_2_(*v*) of a node *v* to describe its local properties in the network; and finally, the combined strategy of Den_1_, Den_2_, BC and IDC is developed to improve the prediction precision. The experimental results demonstrate that LBCC outperforms traditional topological measures for predicting essential proteins, including degree centrality (DC), BC, subgraph centrality (SC), eigenvector centrality (EC), network centrality (NC), and the local average connectivity-based method (LAC). LBCC also improves the prediction precision by approximately 10 percent on the YMIPS and YMBD datasets compared to the most recently developed method, LIDC.

## Introduction

Essential proteins are indispensable to the viability or reproduction of an organism and play a decisive role in cellular life [1]. Deletion of a single essential protein is sufficient for causing lethality or infertility [2]. Compared to non-essential proteins, essential proteins are more likely to be conserved in biological evolution [3]. Essential proteins provide insights into the molecular mechanisms of an organism at the system level, with significant implications for drug design and disease study [4]. For example, in drug development, essential proteins are excellent targets for potential new drugs and vaccines to treat and prevent diseases and for improved diagnostic tools more reliably to detect infections [5].

There are two types of methods for predicting essential proteins. One is experimental procedures, such as RNA interference [6], single gene knockouts [7], and conditional knockouts [8]. However, these experimental procedures require considerable time and resources, even for well-studied organisms, and they are not always practical. The other type of method is bioinformatics computational approaches that take advantage of the abundance of experimental data available for protein interaction networks, such as degree centrality (DC) [9], betweenness centrality (BC) [10], subgraph centrality (SC) [11], eigenvector centrality (EC) [12], network centrality (NC) [13], and the local average connectivity-based method (LAC) [14]. Obviously, the latter is faster and less expensive than the former.

In 2015, Luo and Qi [15] proposed a method named LIDC for discovering essential proteins based on the local interaction density and protein complexes. The experimental results obtained with the YMIPS dataset demonstrated that the performance of LIDC was superior to that of nine reference methods (i.e., DC, BC, NC, LID [15], PeC [16], CoEWC [17], WDC [18], ION [19], and UC [20]).

However, methods based on bioinformatics computational approaches are sensitive to the local or global topological properties of the network, and the prediction precision for identifying essential proteins requires further improvement. In this paper, we first introduce the densities *Den*_1_(*v*) and *Den*_2_(*v*) of a node *v* to describe its local properties in the network. Then, a novel method called LBCC is proposed, which is combined with Den_1_, Den_2_, BC, and IDC, where the local and global properties of the node are measured by Den_1_ and Den_2_ and by BC, respectively, and the information of the protein complex is measured by IDC, which was first introduced in [15]. This combination of features has not previously been considered for this problem.

We performed several experiments on different PPI (protein-protein interaction) networks of *Saccharomyces* *cerevisiae*, YMIPS, YMBD, YHQ and YDIP, which will be described in the Experimental data section. The experimental results demonstrate that our LBCC method provides superior prediction performance compared to centrality measures, including DC, BC, SC, EC, NC, and LAC. In particular, compared to the most recent method, LIDC, which is a more effective method for predicting essential proteins, LBCC improves the prediction precision by at least 10 percent on the YMIPS and YMBD datasets.

## Methods

### Notation

For an undirected simple graph *G*(*V*, *E*) with a set of nodes *V* and a set of edges *E*, a node *v* ∈ *V* denotes a protein and an edge *e*(*u*, *v*)∈*E* denotes an interaction between two proteins *u* and *v*. *N*_*v*_ denotes the set of nodes containing all the neighbors of node *v*, and |*N*_*v*_| denotes the number of nodes in *N*_*v*_. Let *G*[*S*] denote the subgraph of *G* induced by the node set *S*.

### Centrality measures

Many researchers have found that it is significative to predict essential proteins by centrality measures [21, 22]. A PPI network is always represented as an undirected simple graph *G*(*V*, *E*). Here, we will introduce six classical centrality measures based on network topological properties.

*Degree centrality*(*DC*). The degree centrality of a node *v* is the number of its neighbor nodes,

$$\begin{array}{c} DC(v)=deg(v), \end{array}$$

where *deg*(*v*) is the number of its neighbor nodes.

*Betweenness centrality*(*BC*). The betweenness centrality of a node *v* is denoted as the average fraction of the shortest paths passing through the node *v*,

$$\begin{array}{c} BC\left(v\right)=\sum_{s\neq t\neq v\in V}\frac{\sigma_{st}\left(v\right)}{\sigma_{st}}, \end{array}$$

where *σ*_*st*_ is the number of shortest paths between *s* and *t*, and *σ*_*st*_(*v*) is the number of such paths passing through *v*.

*Subgraph centrality*(*SC*). The subgraph centrality of a node *v* accounts for the participation of *v* in all subgraphs of the network,

$$\begin{array}{c} SC\left(v\right)=\sum_{k=0}^{\infty }\frac{\mu_{k}\left(v\right)}{k!}, \end{array}$$

where *μ*_*k*_(*v*) is the number of subgraphs from node *v* to node *v* with length *k*.

*Eigenvector centrality*(*EC*). The eigenvector centrality of a node *v* is the value of the *v*th component of the principal eigenvector of *A*,

$$\begin{array}{c} EC\left(v\right)=\alpha_{max}\left(v\right), \end{array}$$

where *α*_*max*_ represents the eigenvector that corresponds to the largest eigenvalue of the adjacency matrix *A* and *α*_*max*_(*v*) is the *v*th component of *α*_*max*_.

*Local average connectivity centrality*(*LAC*). The local average connectivity centrality of a node *v* is denoted as the local connectivity of its neighbors,

$$\begin{array}{c} LAC\left(v\right)=\frac{\sum_{u\in N_{v}}deg^{C_{v}}\left(u\right)}{|N_{v}|}, \end{array}$$

where *C*_*v*_ is the subgraph *G*[*N*_*v*_] and *deg*^*C*_*v*_^(*u*) is the number of its neighbors in *C*_*v*_ for a node *u* ∈ *N*_*v*_.

*In*-*degree centrality of complex*(*IDC*). The in-degree centrality of complex of a node *v* is denoted as

$$\begin{array}{c} IDC\left(v\right)=\sum_{i\in ComplexSet(v)}IN-Degree{\left(v\right)}_{i}, \end{array}$$

where *ComplexSet*(*v*) represents the set of protein complexes including protein *v* and *IN*-*Degree*(*v*)_*i*_ is represented as the value of *DC*(*v*) for the *i*th protein complex belonging to *ComplexSet*(*v*).

### Local properties of nodes in a PPI network

There are many local properties of nodes in a PPI network, such as the degree centrality (DC) and local clustering coefficient [23], which is defined as

$$\begin{array}{c} LCC\left(v\right)=\frac{2(|E\left(H\right)|-|N_{v}|)}{|N_{v}\left|(\right|N_{v}\left|-1\right)}. \end{array}$$

In this section, we propose two types of local properties of nodes in a PPI network, *Den*_1_(*v*) and *Den*_2_(*v*), which are defined as follows.

*Den*_1_(*v*). For a node *v*, let *H* denote the subgraph of *G*[*N*_*v*_ ∪ {*v*}]; then, we define

$$\begin{array}{c} Den_{1}\left(v\right)=\frac{2|E(H)|}{\left|V(H)|(|V(H)|-1\right)}, \end{array}$$

which is the proportion of the number of the edges to the number of all possible edges of *H*. *Den*_1_(*v*) is somewhat different from *LCC*(*v*), and their relationship is

$$\begin{array}{c} Den_{1}\left(v\right)=\frac{(|N_{v}|-1)LCC\left(v\right)+2}{\left(|N_{v}|+1\right)}. \end{array}$$

**Fig 1:**
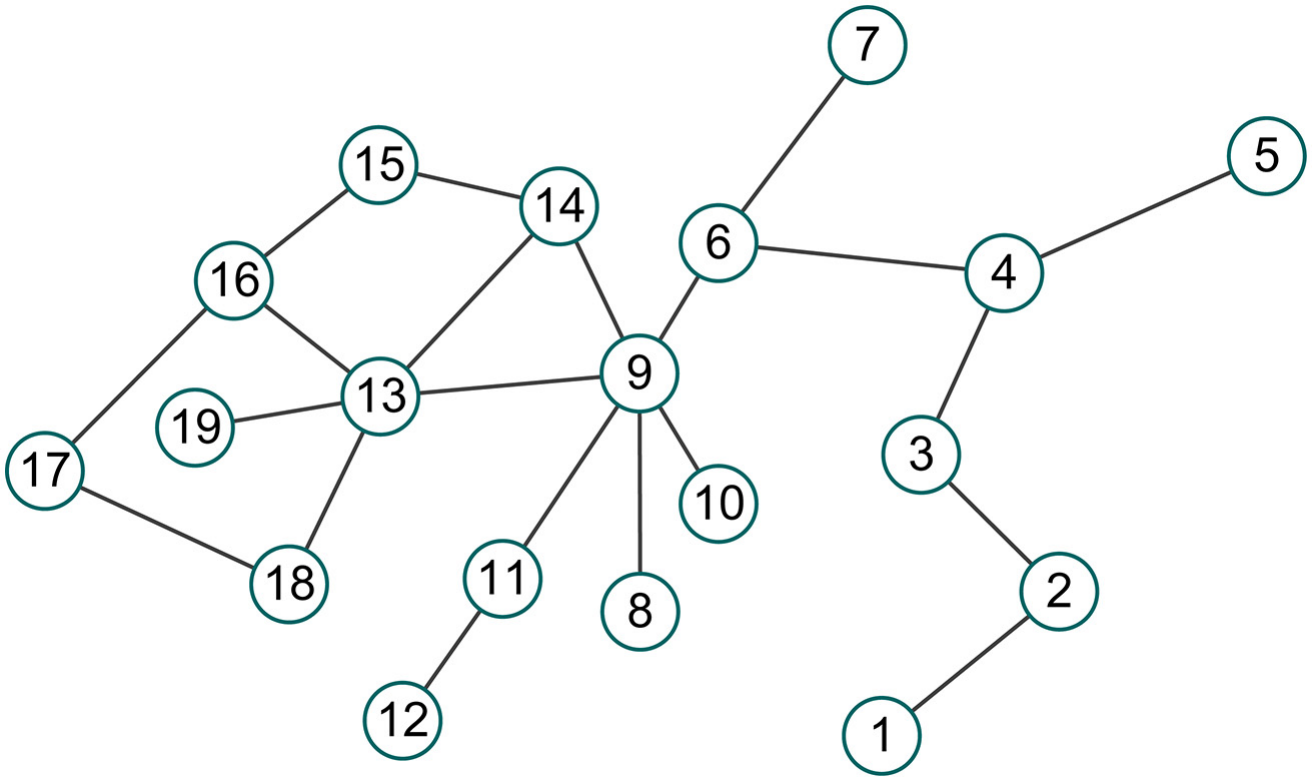
Graph *G*.

*Den*_2_(*v*). For nodes *v* and *u* ∈ *N*_*v*_, let *M*_*u*_ = ⋃_*u* ∈ *N*_*v*__ *N*_*u*_, and let *H* denote the subgraph of *G*[*M*_*u*_ ∪ *N*_*v*_ ∪ {*v*}]; then, we define

$$\begin{array}{c} Den_{2}\left(v\right)=\frac{2|E(H)|}{\left|V(H)|(|V(H)|-1\right)}, \end{array}$$

where *M*_*u*_ is the set of nodes for which the distance to *v* is 2. Hence, *Den*_2_(*v*) is the density of the subgraph induced by *v* and the set of nodes for which the distance to *v* is 1 or 2. Considering the graph *G* shown in Fig 1 as an example, except for the leaf nodes, the values of *Den*_1_(*v*) and *Den*_2_(*v*) of the other nodes are presented in Table 1.

**Table 1:** The values of *Den*_1_(*v*) and *Den*_2_(*v*) of the nodes in graph *G*.

Node	2	3	4	6	9	11	13	14	15	16	17	18
*Den*_1_(*v*)	0.667	0.667	0.500	0.500	0.333	0.667	0.400	0.667	0.667	0.500	0.667	0.667
*Den*_2_(*v*)	0.500	0.333	0.286	0.200	0.165	0.286	0.212	0.218	0.467	0.357	0.500	0.381

To evaluate the effects of Den_1_ and Den_2_ on the prediction of essential proteins, we performed some experiments on the YMIPS and YMBD datasets, which are described in the next section. Consider that the values of BC can represent the global properties of nodes. We first compute the value of *BC*(*v*) of each node *v* in YMIPS and YMBD, and we compute their local properties *Den*_1_(*v*) and *Den*_2_(*v*). For YMIPS, we find that there are 33 pairs of nodes, in which each pair has the same value of *BC*(*v*), and *Den*_1_(*v*) and *Den*_2_(*v*) can facilitate identifying the essential proteins in 6 pairs. For YMBD, we also find that there are 39 pairs of nodes, in which each pair has the same value of *BC*(*v*), and *Den*_1_(*v*) and *Den*_2_(*v*) can facilitate locating the essential proteins in 8 pairs. In Table 2, we list the values of *Den*_1_(*v*), *Den*_2_(*v*) and *BC*(*v*) of these pairs of nodes for YMIPS and YMBD. Hence, we believe that the local properties *Den*_1_(*v*) and *Den*_2_(*v*) are important for aiding in locating essential proteins.

**Table 2:** The values of *Den*_1_(*v*), *Den*_2_(*v*) and *BC*(*v*) for YMIPS and YMBD.

Dataset	Protein	*Den*_1_(*v*)	*Den*_2_(*v*)	*BC*(*v*)	Essentiality status
YMIPS	YNL012W	0.667	0.400	21895	Nonessential
	YPR085C	0.667	0.077	21895	Essential
	YHR004C	0.333	0.024	13528	Nonessential
	YJR112W	0.279	0.027	13528	Essential
	YCL031C	0.500	0.119	8765	Essential
	YCR059C	0.500	0.007	8765	Nonessential
	YDR160W	0.667	0.400	8764	Essential
	YNR019W	0.667	0.250	8764	Nonessential
	YAL025C	0.667	0.116	4383	Essential
	YAL042W	0.667	0.076	4383	Nonessential
	YDR380W	0.667	0.667	1	Nonessential
	YER009W	0.667	0.500	1	Essential
YMBD	YDR180W	0.400	0.099	10670	Essential
	YMR312W	0.400	0.409	10670	Nonessential
	YMR153W	0.667	0.162	10665	Nonessential
	YPR088C	0.762	0.071	10665	Essential
	YEL013W	0.400	0.103	6408	Nonessential
	YPL169C	0.400	0.084	6408	Essential
	YPL204W	0.700	0.311	6405	Essential
	YEL036C	0.667	0.467	6405	Nonessential
	YER029C	0.500	0.364	4273	Essential
	YHR171W	0.500	0.333	4273	Nonessential
	YER167W	0.667	0.222	4272	Nonessential
	YOL034W	0.667	0.427	4272	Essential
	YOR319W	0.667	0.615	2137	Essential
	YIL139C	0.667	0.154	2137	Nonessential
	YLR342W	0.833	0.137	1068	Nonessential
	YHR172W	0.833	0.179	1068	Essential

### New centrality measure: LBCC

In this section, we propose a new method, LBCC, by combining Den_1_, Den_2_, BC and IDC. The following basic concepts underlie LBCC:

1. essential proteins tend to form highly connected clusters [24];
2. essential proteins gather in protein complexes [20]; and
3. both local and global properties are important for aiding in locating essential proteins.

Therefore, for a node *v* of the network, we use *IDC*(*v*) to represent its information on protein complexes and *BC*(*v*) to represent its global properties. For the contribution of local properties and highly connected clusters, we use *Den*_1_(*v*) and *Den*_2_(*v*). Because the value ranges of these measures differ, we apply a log transformation to normalize the data.

Now, we can describe our new measurement LBCC for evaluating the essentiality of a node *v*,

$$\begin{array}{c} LBCC\left(v\right)=a*\log Den_{1}\left(v\right)+b*\log Den_{2}\left(v\right)+c*\log IDC\left(v\right)+d*\log BC\left(v\right), \end{array}$$

where *a*, *b*, *c*, and *d* are scaling parameters that range from 0 to 10 and represent the importance of the corresponding item used in the LBCC calculation. We set *IDC*(*v*) = 0.001 if a protein *v* does not appear in any protein complex.

We perform a large number of experiments to identify essential proteins in the YMIPS dataset, and we find that the measurement LBCC has the best performance when a, b, c and d are set to 1, 4, 3 and 1, respectively. To improve the values of these parameters, we also conduct some experiments using a logistic regression classifier; however, the results are extremely poor due to the imbalanced datasets, in which the number of nonessential proteins is approximately three times greater than the number of essential proteins for the four PPI networks.

As shown in Table 2, the values of BC are far greater than those of Den_1_ and Den_2_. For IDC, the majority of its values are between 10 and 100 on the YMIPS dataset. Hence, IDC and BC are more important than Den_1_ and Den_2_ when calculating LBCC.

## Results and Discussion

### Experimental data

To evaluate the performance of the LBCC method, we used *Saccharomyces* *cerevisiae* as the experimental material because relatively reliable and complete PPI data are available for this organism. The PPI network data are from the MIPS database (Mammalian Protein-Protein Interaction Database) [25], the DIP database [26], and other datasets from the website of the Mark Gerstein Lab (gersteinlab.org).

We selected four different datasets. The first dataset, a MIPS dataset, was marked YMIPS (S1 Text); the second and third datasets from the Mark Gerstein Lab were marked YMBD (S2 Text) and YHQ (S3 Text), respectively; and the fourth dataset, a DIP dataset, was marked YDIP (S4 Text). YMIPS included 4546 proteins and 12319 interactions, and its average degree was approximately 5.42. YMBD, which was selected from MIPS, BIND and DIP, includes 2559 proteins and 11835 interactions, and its average degree was approximately 9.25. YHQ was constructed by Yu *et al.* [27] comprehensively and reliably and includes 4743 proteins and 23294 interactions in total. The average degree of YHQ was approximately 9.82. YDIP included 5093 proteins and 24743 interactions, and its average degree was approximately 9.72.

The essential proteins (S1 Excel) of *Saccharomyces* *cerevisiae* were collected from the following databases: MIPS [25], SGD (Saccharomyces Genome Database) [28], DEG (Database of Essential Genes) [29], and SGDP (Saccharomyces Genome Deletion Project) [2]. Detailed information on the datasets is presented in Table 3.

**Table 3:** Information on the four PPI datasets: YMIPS, YMBD, YHQ and YDIP.

Dataset	Proteins	Interactions	Average degree	Essential proteins
YMIPS	4546	12319	5.42	1016
YMBD	2559	11835	9.25	763
YHQ	4743	23294	9.82	1108
YDIP	5093	24743	9.72	1167

The protein complex set (S2 Excel) was directly obtained from [15] and contained 745 protein complexes (comprising 2167 proteins) from four protein complex datasets: CM270 [25], CM425 [30], CYC408 and CYC428 [31, 32]. All data and our code are available at website https://github.com/qindynasty/LBCC.

### Evaluation methods

In general, several statistical measures, such as sensitivity (SN), specificity (SP), positive predictive value (PPV), negative predictive value (NPV), F-measure (F), and accuracy (ACC), are used to determine how effectively the essential proteins are identified by different methods (see the references [13, 15]). We introduce them in this section to evaluate the effectiveness of the proposed method LBCC. First, we provide four statistical terms:

- *True positives*(*TP*). The essential proteins that are correctly selected as essential.
- *False positives*(*FP*). The nonessential proteins that are incorrectly selected as essential.
- *True negatives*(*TN*). The nonessential proteins that are correctly selected as nonessential.
- *False negatives*(*FN*). The essential proteins that are incorrectly selected as nonessential.

Next, we provide the definitions of six statistical measures:

*Sensitivity*. Sensitivity is the ratio of the proteins that are correctly selected as essential to the total number of essential proteins,

$$\begin{array}{c} SN=\frac{TP}{TP+FN}, \end{array}$$

*Specificity*. Specificity is the ratio of the nonessential proteins that are correctly selected as nonessential to the total number of nonessential proteins,

$$\begin{array}{c} SP=\frac{TN}{TN+FP}, \end{array}$$

*Positive predictive value*. Positive predictive value refers to the ratio of the proteins that are correctly selected as essential,

$$\begin{array}{c} PPV=\frac{TP}{TP+FP}, \end{array}$$

*Negative predictive value*. Negative predictive value refers to the ratio of the proteins that are correctly selected as nonessential,

$$\begin{array}{c} NPV=\frac{TN}{TN+FN}, \end{array}$$

*F*-*measure*. F-measure refers to the harmonic mean of *SN* and *PPV*,

$$\begin{array}{c} F=\frac{2*SN*PPV}{SN+PPV}, \end{array}$$

*Accuracy*. Accuracy refers to the ratio of the proteins that are correctly selected as essential and nonessential in all the results,

$$\begin{array}{c} ACC=\frac{TP+TN}{P+N}, \end{array}$$

in which *P* represents the number of essential proteins and *N* represents the number of nonessential proteins.

### Comparison with other prediction measures

To evaluate the performance of LBCC, we compared LBCC and other prediction measures using the four datasets described in the Experimental data section. The compared prediction measures included LIDC, DC, BC, SC, EC, NC, and LAC. The algorithm for LIDC was implemented according to [15], and the other algorithms were implemented using CytoNCA [33], a plugin of Cytoscape for centrality analysis of PPI networks.

**Fig 2:**
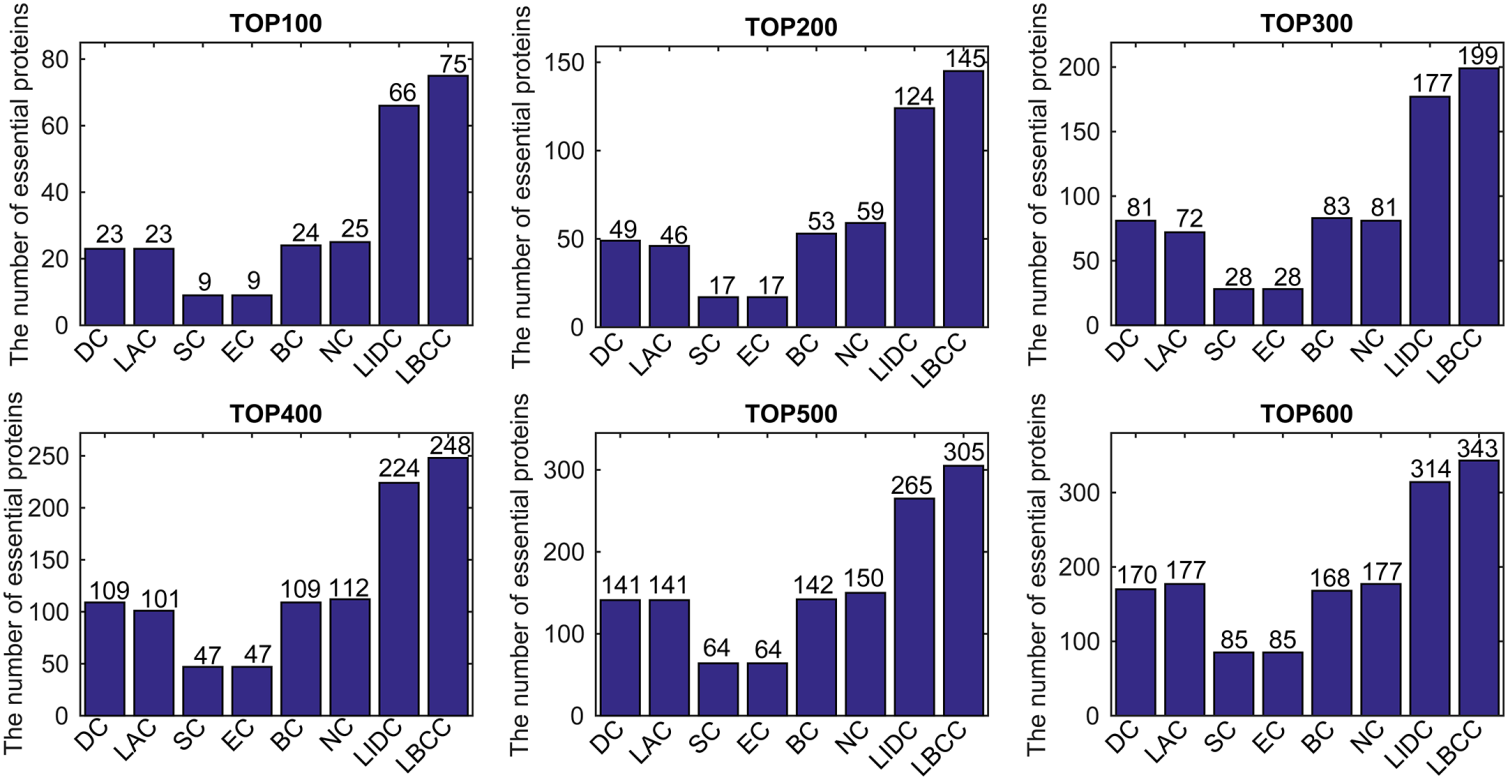
The number of true essential proteins predicted by LBCC and the other seven previously proposed methods for the YMIPS network.

**Fig 3:**
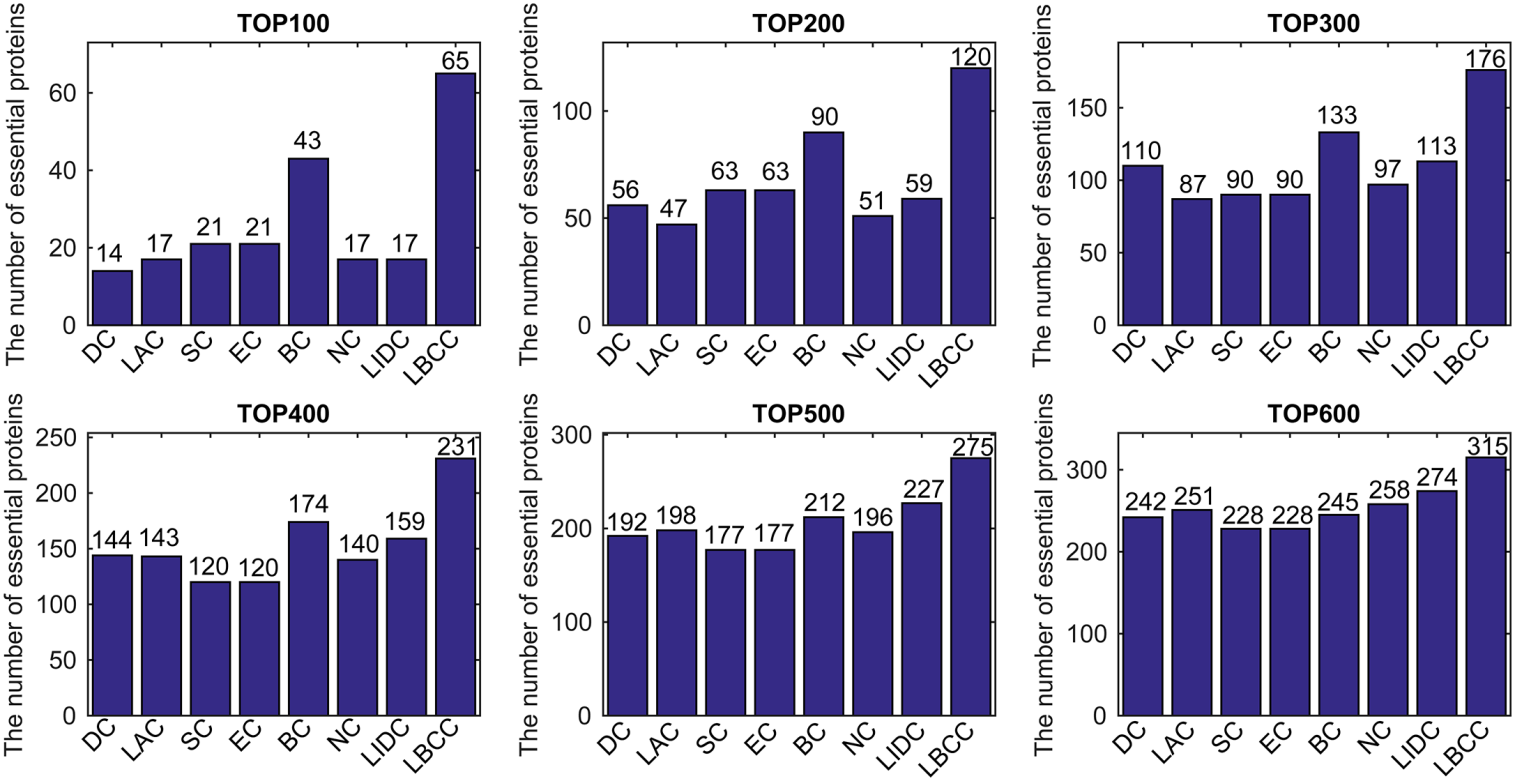
The number of true essential proteins predicted by LBCC and the other seven previously proposed methods for the YMBD network.

**Fig 4:**
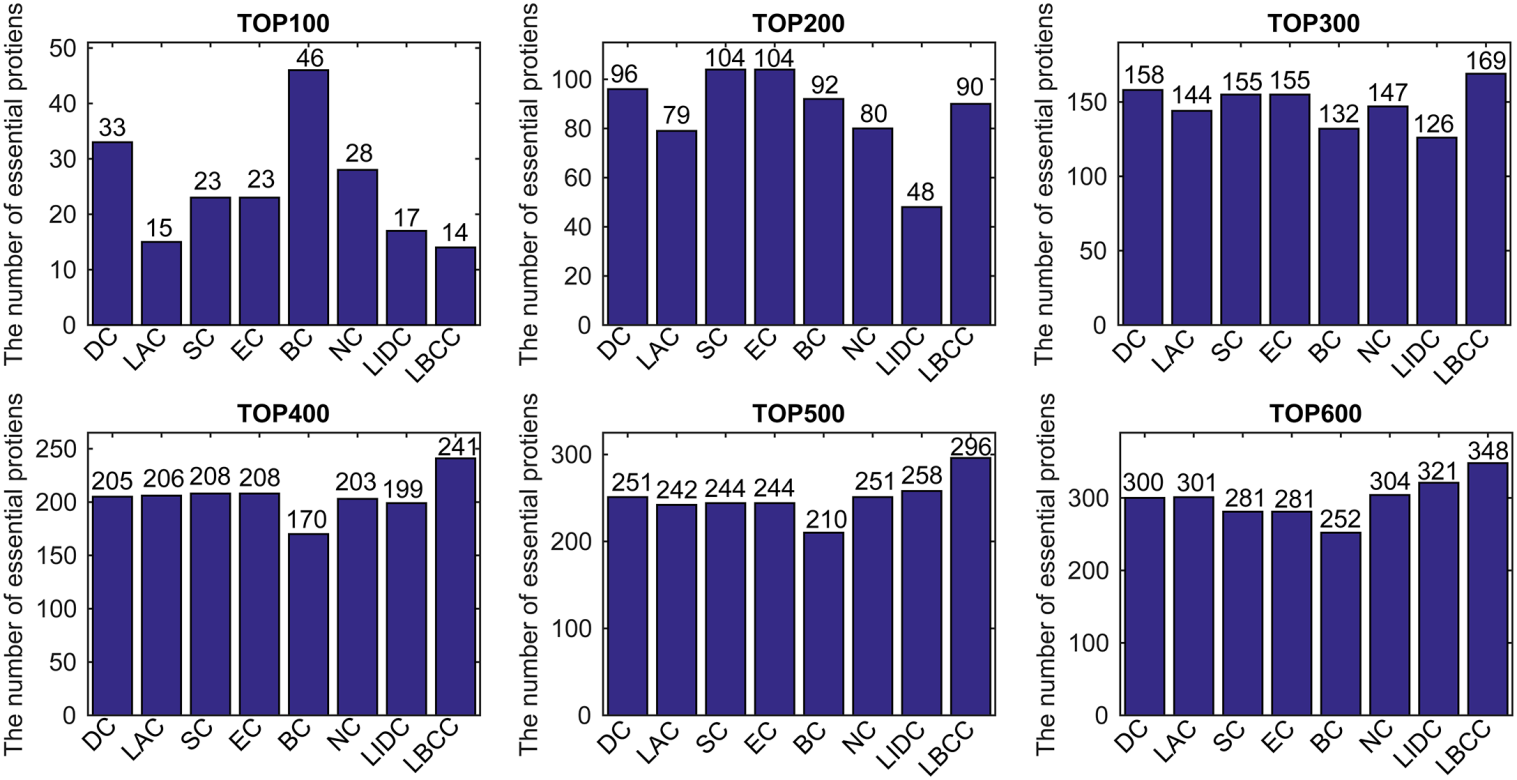
The number of true essential proteins predicted by LBCC and the other seven previously proposed methods for the YHQ network.

**Fig 5:**
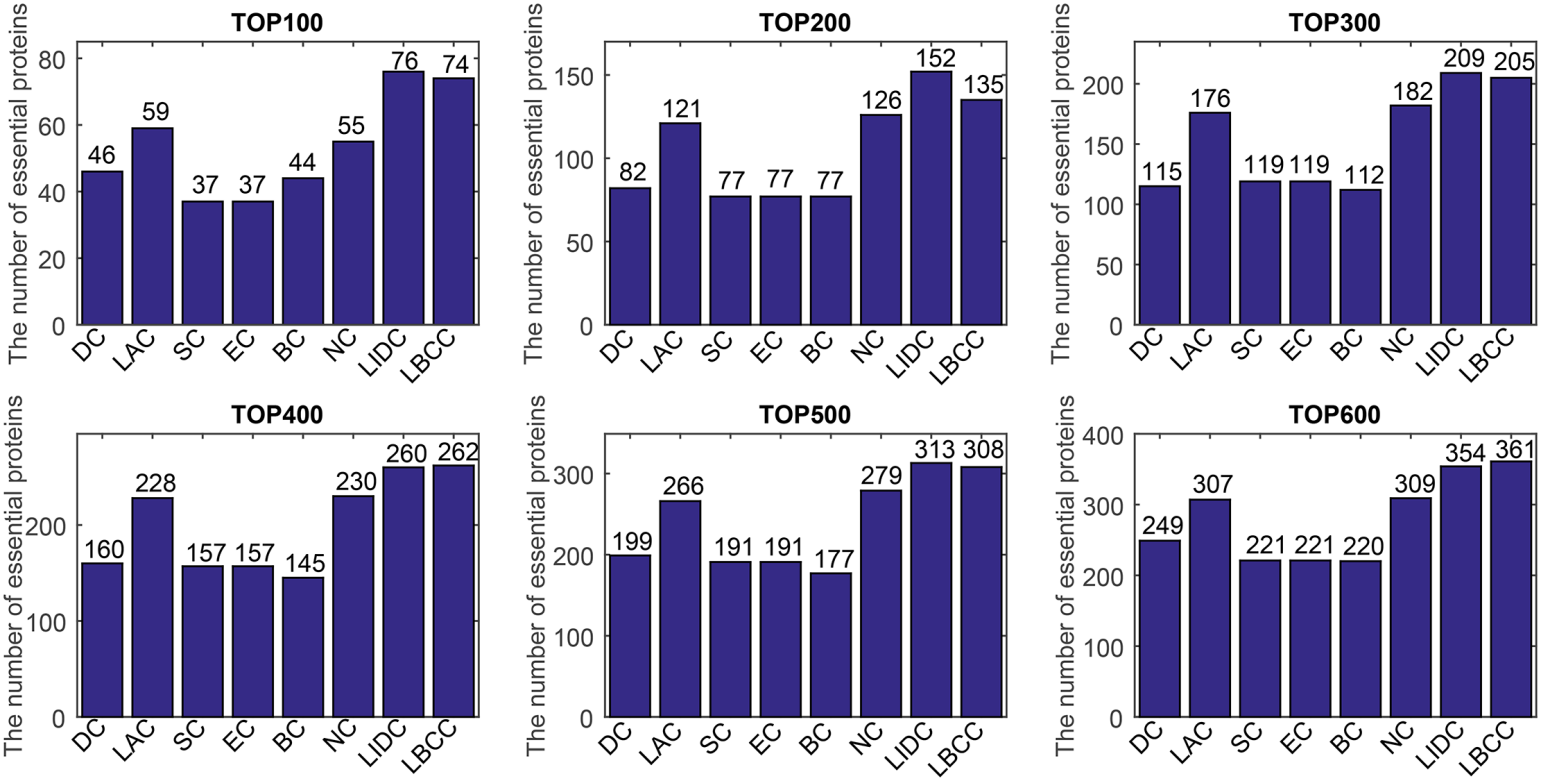
The number of true essential proteins predicted by LBCC and the other seven previously proposed methods for the YDIP network.

First, we ranked proteins in descending order based on their LBCC values and other prediction measures; second, we selected the top 100, 200, 300, 400, 500, and 600 proteins as essential proteins; and finally, the number of true essential proteins was determined. The prediction results of the eight methods for the four different networks are shown in Figs 2–5.

For the YMIPS dataset shown in Fig 2, LIDC, the most recent method, had the best performance, with 66, 124, 177, 224, 265, and 314 true essential proteins identified at six levels from the top 100 to top 600. By comparison, the numbers of true essential proteins predicted by LBCC were 75, 145, 199, 248, 305, and 343, respectively. Compared to LIDC, LBCC exhibited superior performance and increased the prediction precision by more than 13, 16, 12, 10, 15 and 9 percent at six levels from the top 100 to top 600.

For the YMBD dataset shown in Fig 3, except for LBCC, the largest numbers of true essential proteins identified were 43 (BC), 90 (BC), 133 (BC), 174 (BC), 212 (BC), and 258 (NC) at six levels from the top 100 to top 600. By comparison, LBCC identified 65, 120, 176, 231, 275, and 315 true essential proteins, improving the prediction precision by more than 51, 33, 32, 32, 29, and 22 percent at six levels from the top 100 to top 600.

For the YHQ dataset shown in Fig 4, BC achieved the best result at the top 100 level, and SC and EC attained the best results at the top 200 level. At four levels from the top 300 to top 600, LBCC produced the best results, and the numbers of true essential proteins identified were 169, 241, 296 and 348.

For the YDIP dataset shown in Fig 5, LIDC achieved the best results at the top 100, 200, 300 and 500 levels, and LBCC attained the best results at the top 400 and 600 levels. At six levels, the numbers of true essential proteins identified by LIDC were 76, 152, 209, 260, 313, and 354. By comparison, the numbers of true essential proteins identified by LBCC were 74, 135, 205, 262, 308, and 361, respectively. The results predicted by LBCC were similar to those obtained using LIDC at the top 100, 300 and 500 levels.

Thus, our experiments indicate that LBCC can identify more essential proteins than the other methods in most cases.

### Validation using six statistical methods and precision-recall curves

In this section, we compared LBCC and the other seven prediction measures using the six statistical methods described in the Evaluation methods section. We ranked the proteins in descending order based on the values of eight measures and selected the top 20 percent as essential proteins; the remaining proteins were considered nonessential proteins. The results are presented in Table 4, and the values of the six statistical methods for LBCC were consistently higher than those for the other methods on the first two networks, indicating that LBCC can predict essential proteins more accurately. For the YHQ dataset, the results predicted by LBCC were identical to those obtained using LIDC. For the YDIP dataset, the results predicted by LBCC were similar to those obtained using LIDC.

**Table 4:** Comparative analysis of LBCC and the other seven previously proposed methods in terms of SN, SP, PPV, NPV, F-measure, and ACC with four different datasets.

Dataset	Methods	SN	SP	PPV	NPV	F-measure	ACC
YMIPS	DC	0.252	0.815	0.282	0.791	0.266	0.689
	LAC	0.269	0.820	0.300	0.796	0.284	0.697
	SC	0.139	0.782	0.155	0.759	0.146	0.639
	EC	0.139	0.782	0.155	0.759	0.146	0.639
	BC	0.249	0.814	0.278	0.790	0.263	0.688
	NC	0.281	0.824	0.315	0.799	0.297	0.702
	LIDC	0.423	0.864	0.473	0.839	0.447	0.766
	LBCC	**0.430**	**0.866**	**0.481**	**0.841**	**0.454**	**0.769**
YMBD	DC	0.260	0.825	0.387	0.724	0.311	0.657
	LAC	0.271	0.830	0.404	0.728	0.325	0.664
	SC	0.239	0.816	0.355	0.716	0.285	0.644
	EC	0.239	0.816	0.355	0.716	0.285	0.644
	BC	0.283	0.835	0.422	0.733	0.339	0.671
	NC	0.266	0.828	0.396	0.726	0.318	0.660
	LIDC	0.308	0.846	0.459	0.742	0.369	0.685
	LBCC	**0.372**	**0.873**	**0.555**	**0.766**	**0.445**	**0.724**
YHQ	DC	0.401	0.861	0.468	0.825	0.432	0.754
	LAC	0.431	0.870	0.504	0.834	0.465	0.768
	SC	0.326	0.838	0.380	0.803	0.351	0.719
	EC	0.326	0.838	0.380	0.803	0.351	0.719
	BC	0.330	0.840	0.386	0.804	0.356	0.721
	NC	0.426	0.869	0.497	0.832	0.459	0.765
	LIDC	**0.449**	**0.876**	**0.524**	**0.839**	**0.483**	**0.776**
	LBCC	**0.449**	**0.876**	**0.524**	**0.839**	**0.483**	**0.776**
YDIP	DC	0.354	0.846	0.406	0.815	0.378	0.733
	LAC	0.405	0.861	0.465	0.830	0.433	0.757
	SC	0.323	0.837	0.370	0.806	0.345	0.719
	EC	0.323	0.837	0.370	0.806	0.345	0.719
	BC	0.308	0.832	0.354	0.802	0.330	0.712
	NC	0.398	0.859	0.456	0.827	0.425	0.753
	LIDC	0.446	0.873	0.511	0.841	0.476	0.775
	LBCC	**0.446**	**0.873**	**0.512**	**0.841**	**0.477**	**0.776**

The precision-recall curve is a statistical method used for assessing the stability of the eight prediction measures. This curve is obtained by plotting

$$\begin{array}{c} Precision\left(n\right)=\frac{TP(n)}{TP(n)+FP(n)}, \end{array}$$

$$\begin{array}{c} Recall\left(n\right)=\frac{TP(n)}{P}, \end{array}$$

where *TP*(*n*) is the total number of essential proteins correctly identified as essential proteins and *FP*(*n*) is the total number of nonessential proteins incorrectly identified as essential proteins among the top *n* proteins. *P* is the total number of essential proteins under consideration.

**Fig 6:**
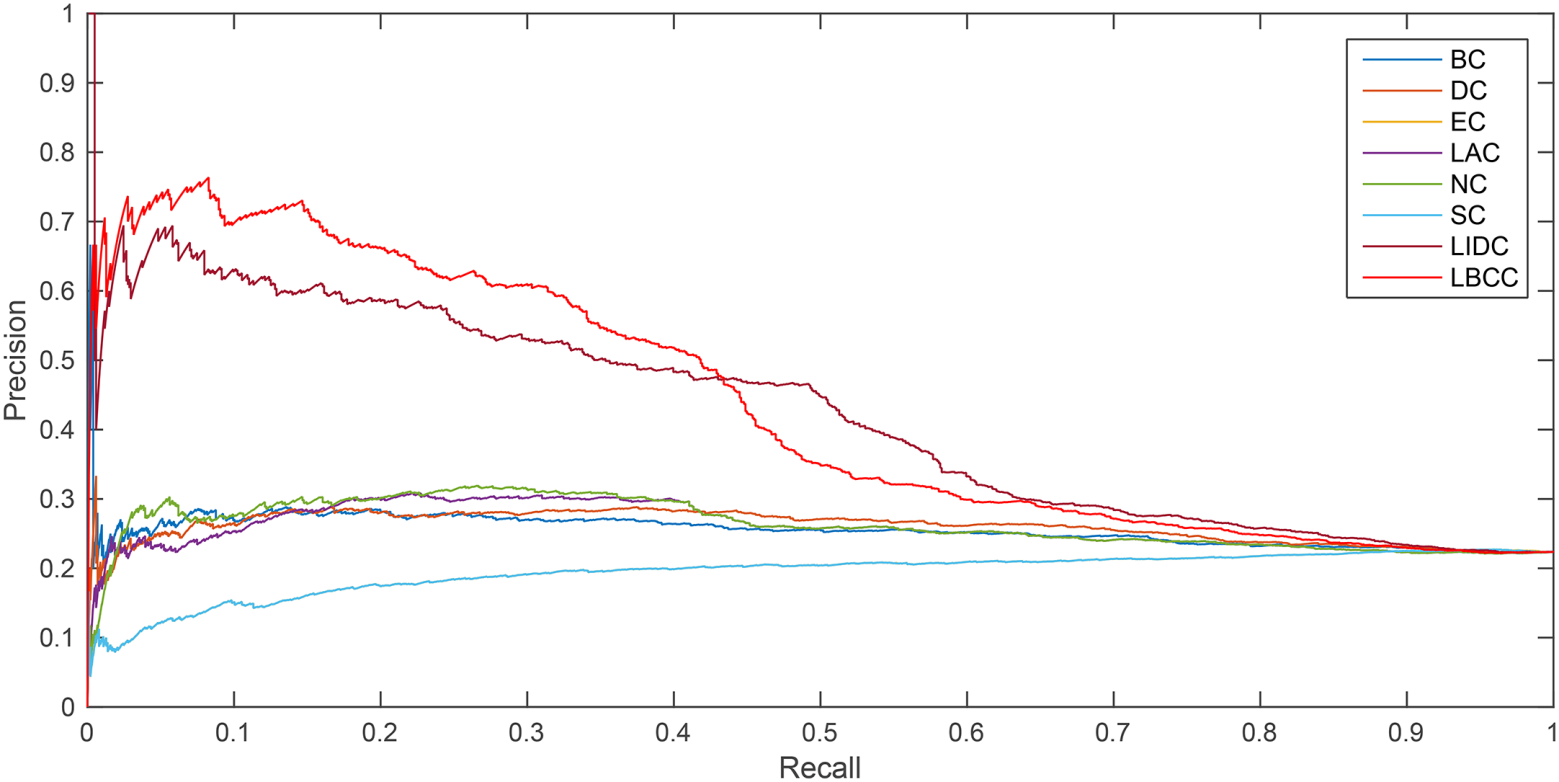
PR curves of LBCC and the other seven previously proposed methods for the YMIPS network.

As shown in Fig 6, LBCC and LIDC performed well for the YMIPS network. The break-even point for the two measures, LIDC and LBCC, at which the curves intersect was 0.46. Between the recall levels of 0 and 0.46, LBCC performed significantly better than LIDC.

**Fig 7:**
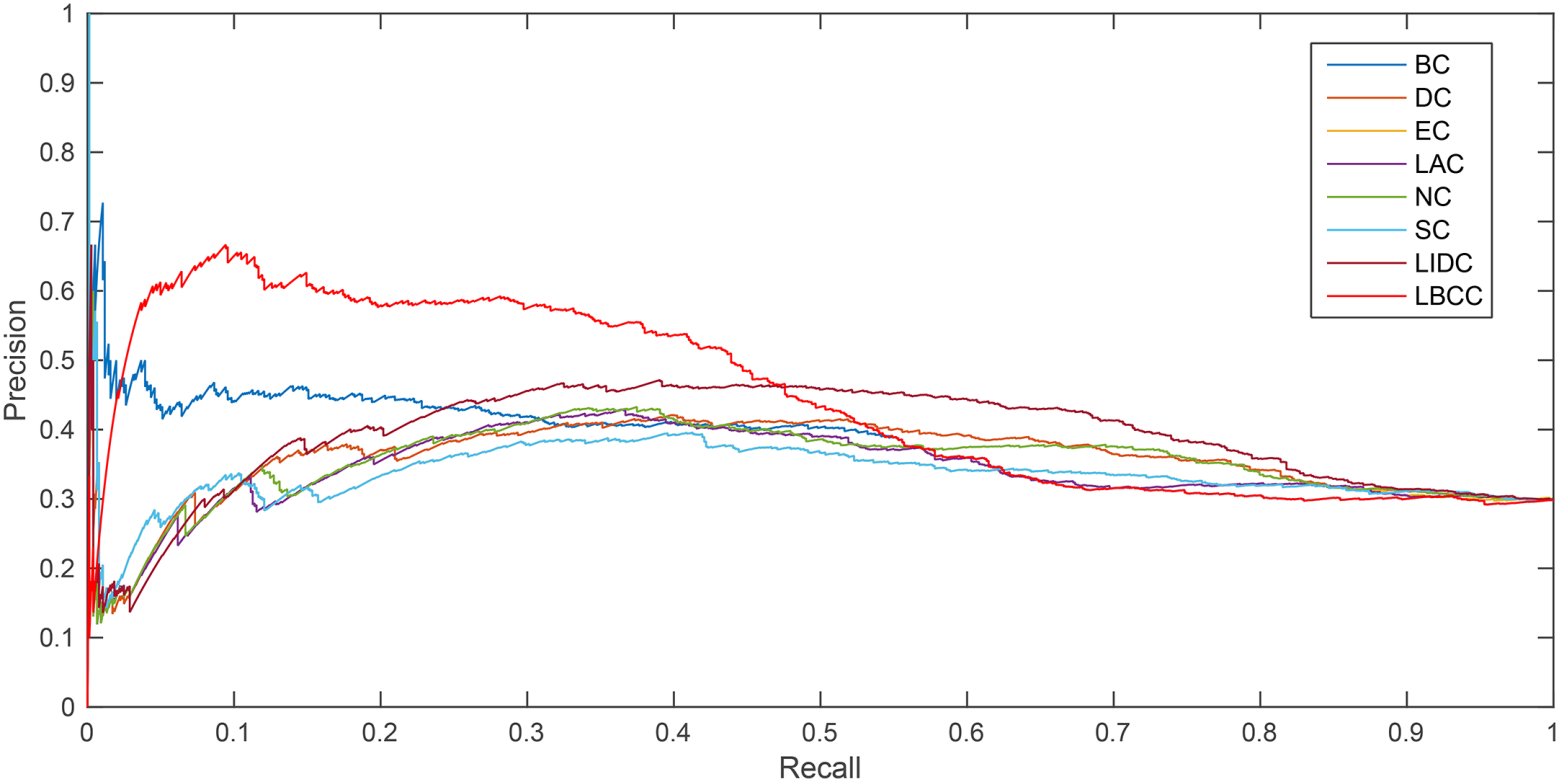
PR curves of LBCC and the other seven previously proposed methods for the YMBD network.

As shown in Fig 7, LBCC performed particularly well for the YMBD network between the recall levels of 0 and 0.46.

**Fig 8:**
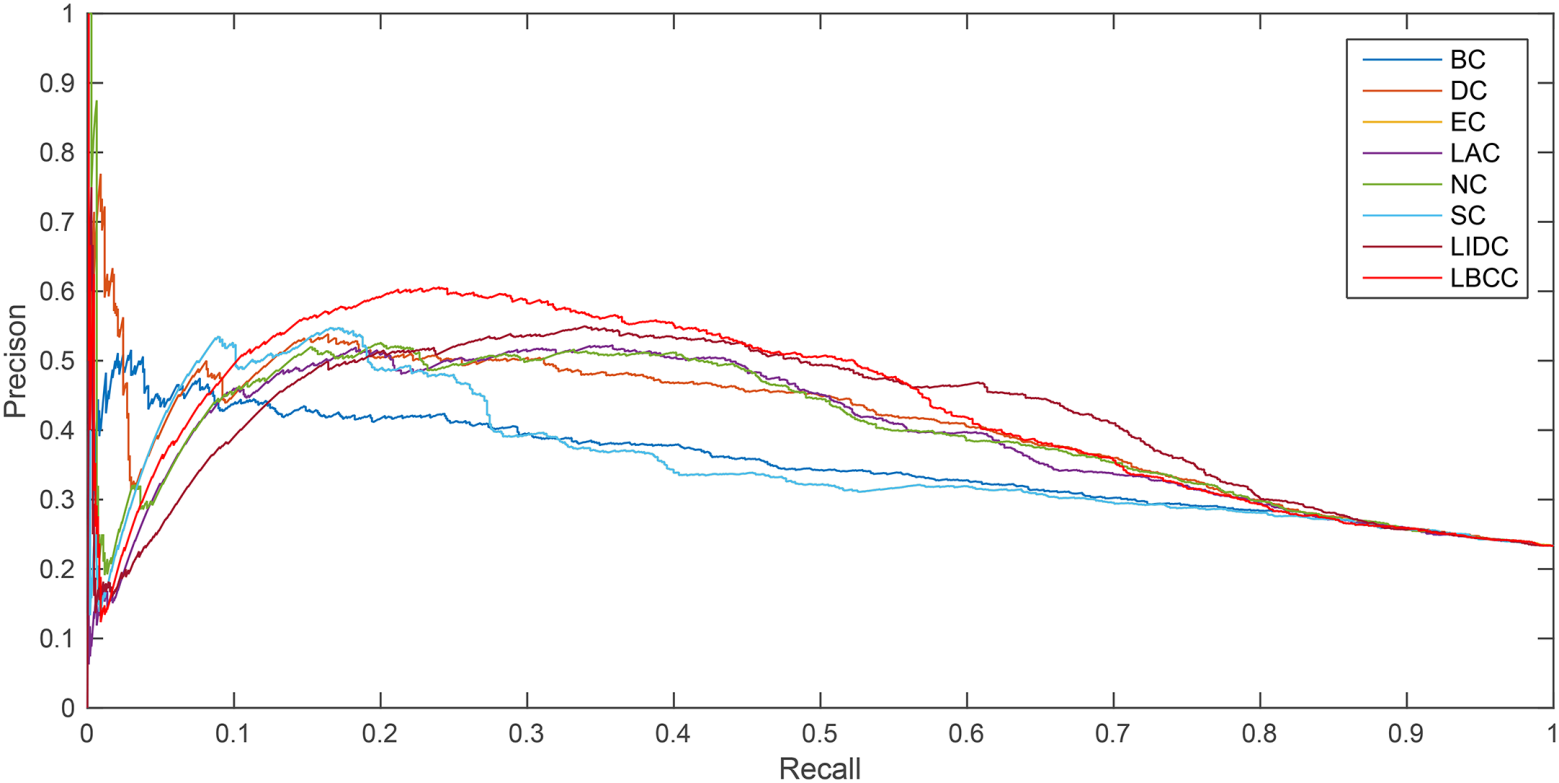
PR curves of LBCC and the other seven previously proposed methods for the YHQ network.

As shown in Fig 8, for the YHQ network, LBCC performed better between the recall levels of 0.1 and 0.56.

**Fig 9:**
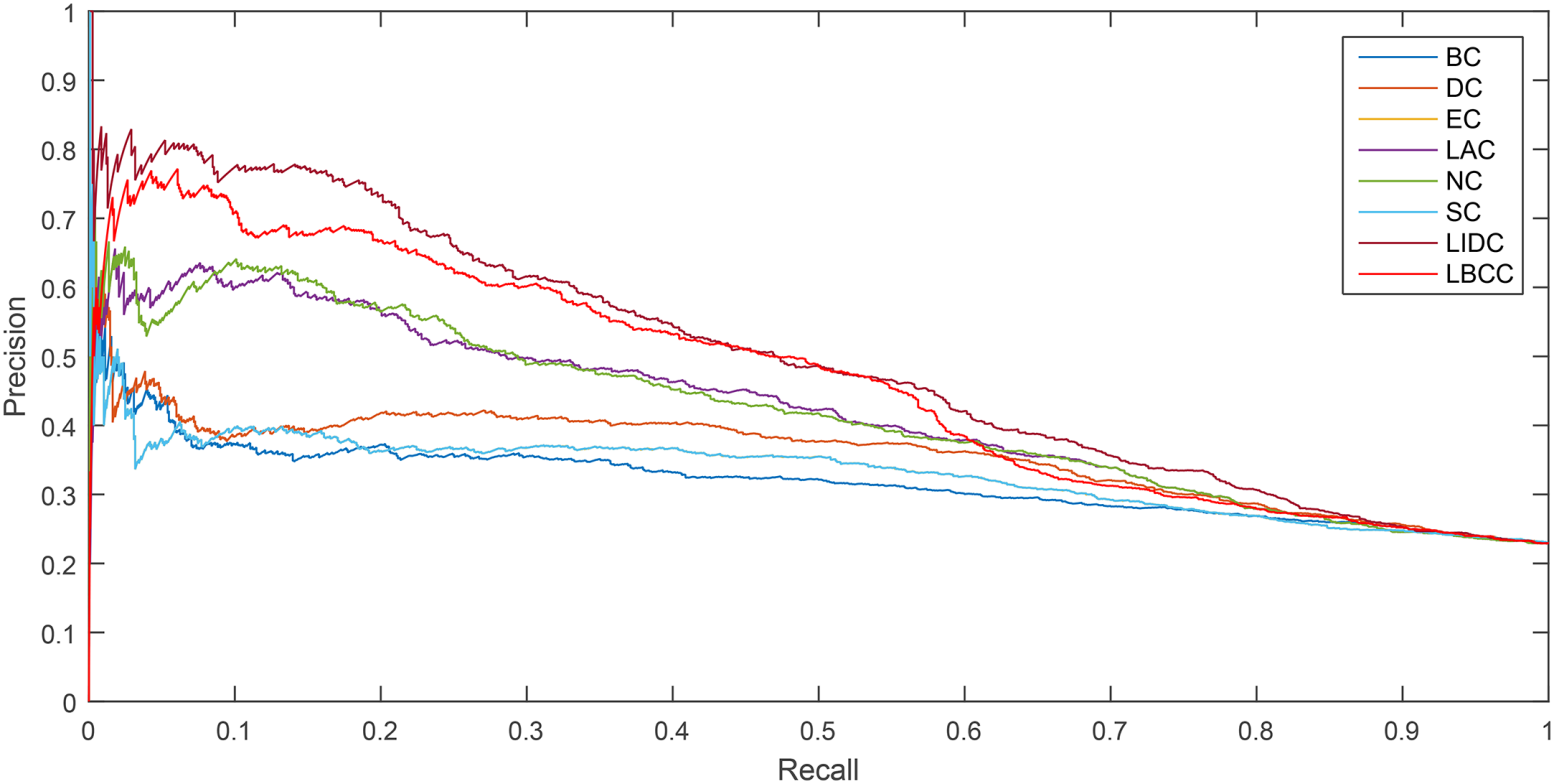
PR curves of LBCC and the other seven previously proposed methods for the YDIP network.

For the YDIP network, as shown in Fig 9, LBCC tended to provide less desirable results compared with LIDC.

The analysis of the six statistical methods and precision-recall curves indicated that LBCC not only has better prediction precisions than the other seven methods but it also delivers more stable performance for the first three networks.

### Validation using jackknife methodology

**Fig 10:**
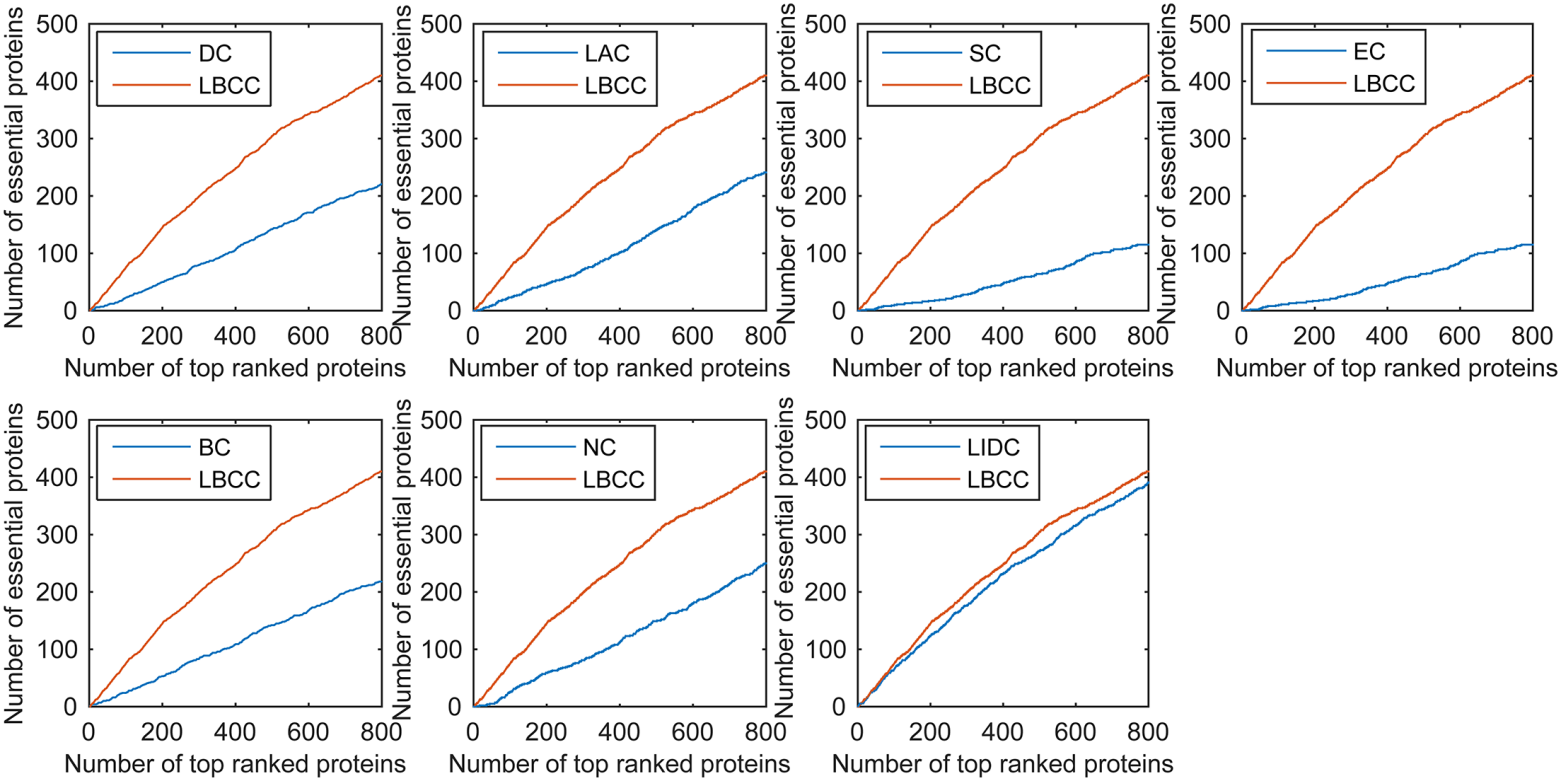
Jackknife curves of LBCC and the other seven previously proposed methods for the YMIPS network.

**Fig 11:**
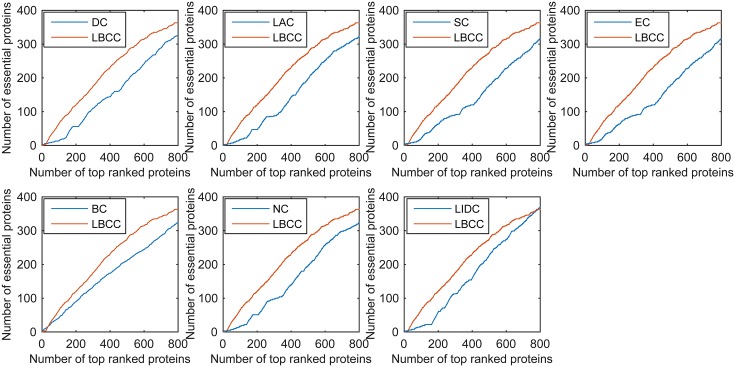
Jackknife curves of LBCC and the other seven previously proposed methods for the YMBD network.

**Fig 12:**
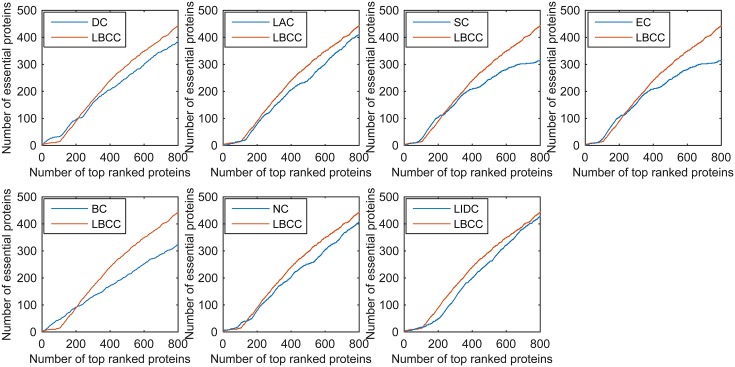
Jackknife curves of LBCC and the other seven previously proposed methods for the YHQ network.

**Fig 13:**
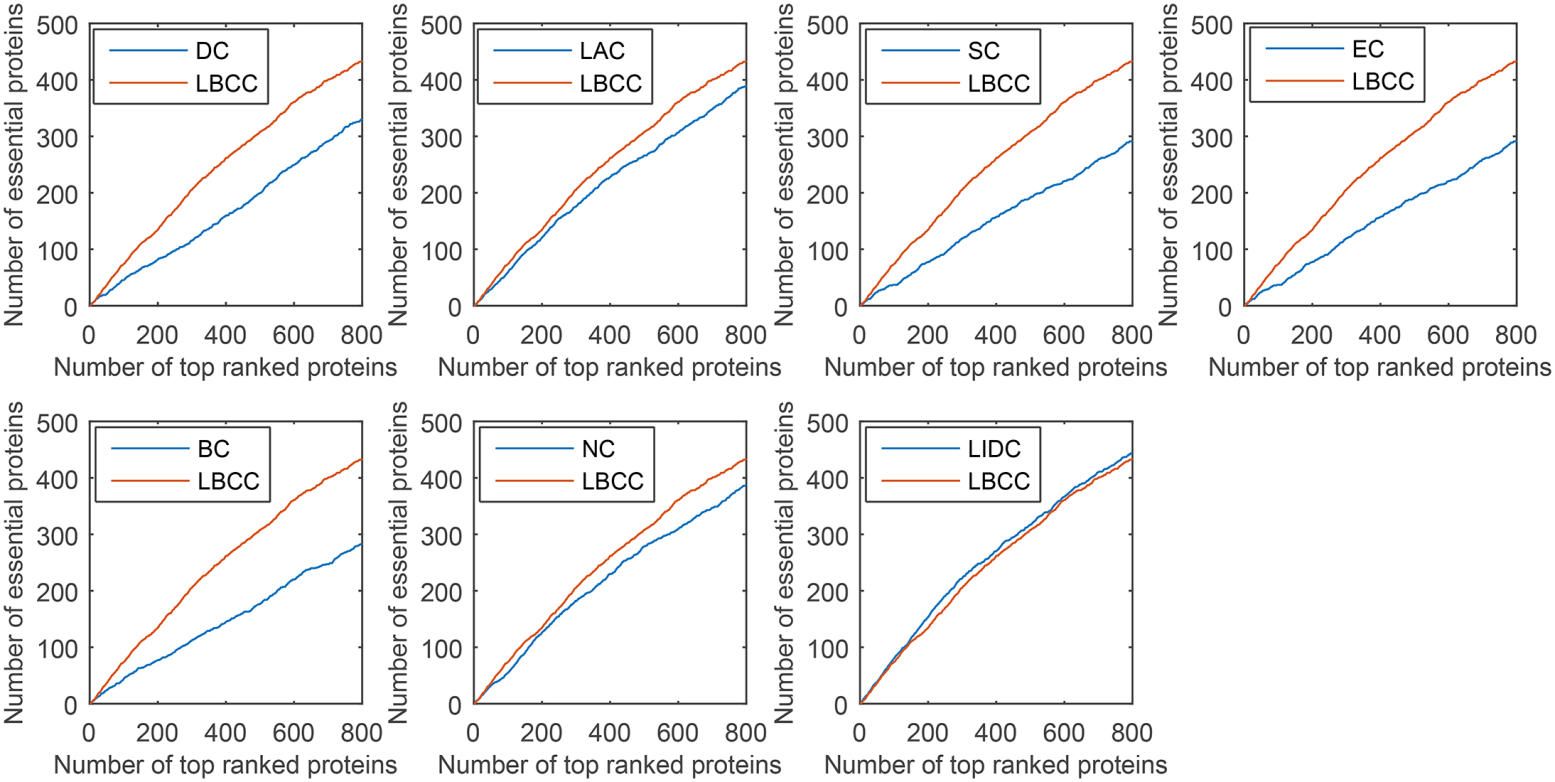
Jackknife curves of LBCC and the other seven previously proposed methods for the YDIP network.

We used the jackknife methodology developed by Holman *et al.* [34] to assess the generality of our trained predictor. First, we ranked the proteins in descending order based on their values obtained using the eight prediction methods. Then, the jackknife curve was plotted according to the cumulative number of the true essential proteins. As shown in Figs 10–13, the *x*-axis represents the proteins ranked in descending order from left to right according to the values computed using the corresponding methods, and the *y*-axis represents the number of true essential proteins among the top *n* proteins, where *n* is the number along the *x*-axis.

As shown in Figs 10–12, the sorted curve of LBCC is significantly better than those of the other prediction measures for the YMIPS, YMBD and YHQ data. For the YDIP network, as shown in Fig 13, LBCC exhibited a performance similar to that of LIDC and superior to those of all the other methods. Hence, the LBCC method is feasible and effective for predicting essential proteins for the first three networks.

### Analysis of the differences between LBCC and other measures

To further determine why LBCC performs well on the four datasets for predicting essential proteins, we studied the difference between LBCC and the other prediction measures by predicting a small number of proteins. Let *A* ∩ *B* denote the set of proteins predicted by both methods A and B, *A* − *B* denote the set of proteins predicted by method A but not by method B, and *A* ∪ *B* denote the set of proteins predicted by method A or B.

We compared the performances of LBCC and the other seven methods in predicting the top 100 proteins ranked by the corresponding methods. The comparison results are presented in Table 5.

**Table 5:** Analysis of the differences between LBCC and the other seven methods in predicting proteins for the YMIPS, YMBD, YHQ and YDIP data.

dataset	measure	|*LBCC* ⋂ *M*|	|*LBCC* − *M*|	true essential proteins in *LBCC* − *M*	true essential proteins in *M* − *LBCC*
YMIPS	DC	1	99	74	22
	LAC	18	82	66	14
	SC	0	100	75	9
	EC	0	100	75	9
	BC	1	99	74	23
	NC	17	83	66	16
	LIDC	35	65	51	41
YMBD	DC	20	80	62	11
	LAC	11	89	62	14
	SC	12	88	62	18
	EC	12	88	62	18
	BC	13	87	59	37
	NC	12	88	62	14
	LIDC	18	82	62	14
YHQ	DC	37	63	7	26
	LAC	36	64	7	8
	SC	36	64	7	16
	EC	36	64	7	16
	BC	5	95	12	42
	NC	37	63	7	21
	LIDC	48	52	5	8
YDIP	DC	4	96	70	42
	LAC	28	72	53	38
	SC	0	100	74	37
	EC	0	100	74	37
	BC	4	96	70	40
	NC	23	77	58	39
	LIDC	66	34	21	27

**Fig 14:**
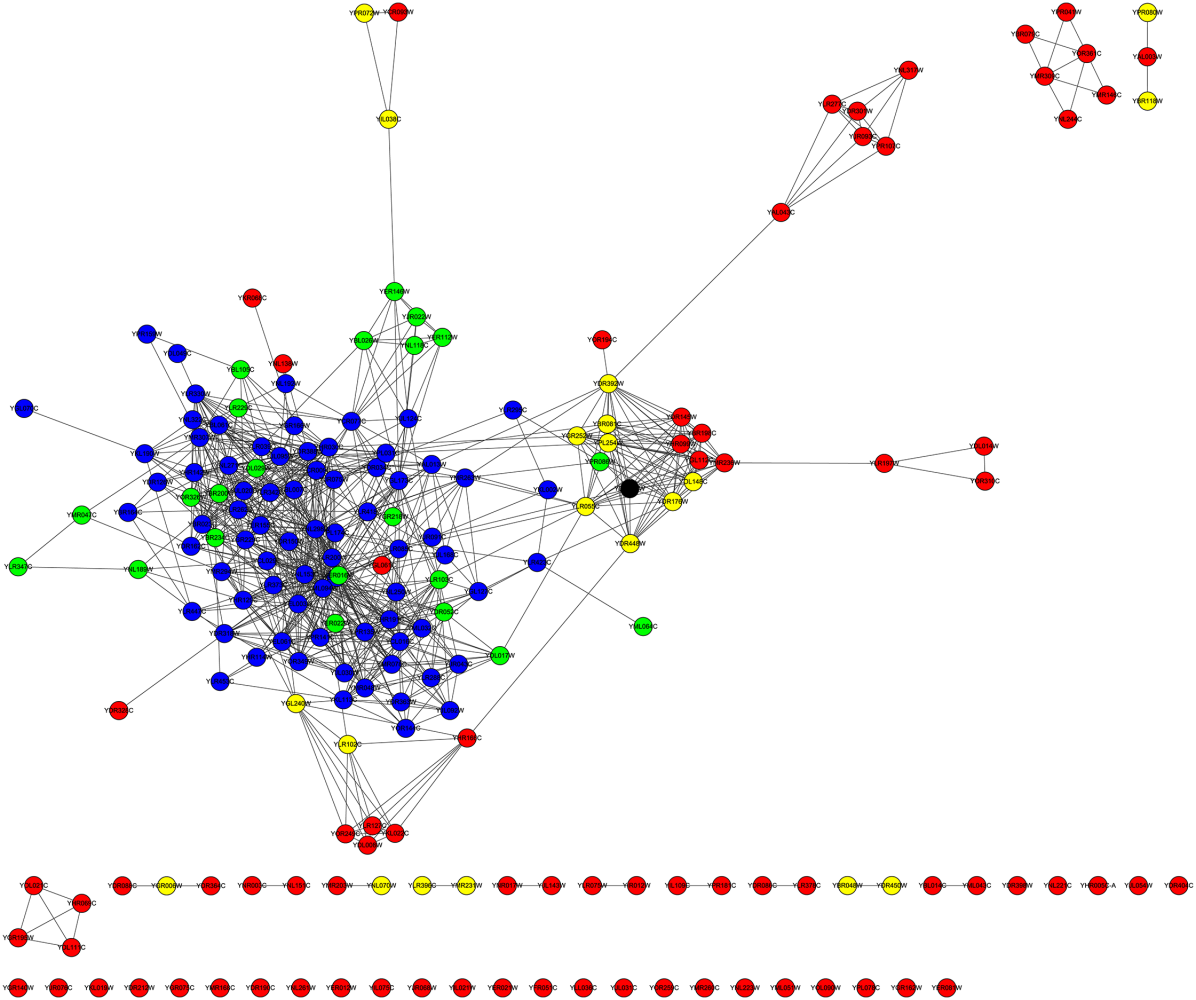
The top 199 proteins in the YMIPS network identified by DC ∪ LBCC. The green nodes and blue nodes are proteins identified by *DC*; the former are true essential proteins, and the latter are nonessential proteins. The red nodes and yellow nodes are proteins identified by *LBCC*; the former are true essential proteins, and the latter are nonessential proteins. The black nodes are the overlapping proteins.

**Fig 15:**
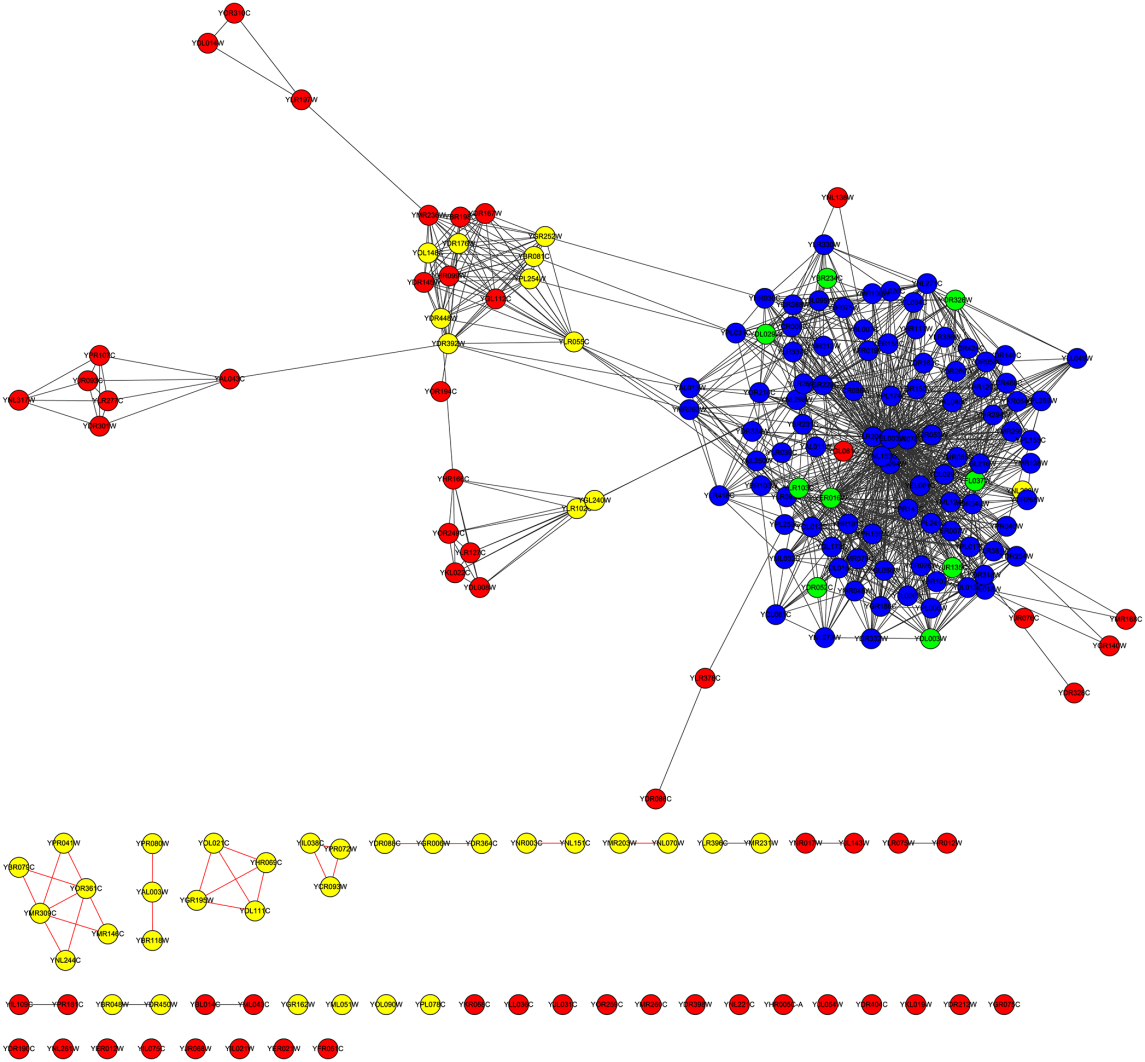
The top 200 proteins in the YMIPS network identified by SC ∪ LBCC. The green nodes and blue nodes are proteins identified by *SC*; the former are true essential proteins, and the latter are nonessential proteins. The red nodes and yellow nodes are proteins identified by *LBCC*; the former are true essential proteins, and the latter are nonessential proteins.

For the YMIPS dataset, as indicated in column |*LBCC* ∩ *M*|, the rates of overlap of the proteins predicted by LBCC and the other six methods (DC, LAC, SC, EC, BC, and NC) were less than 20 percent, and no protein was predicted by LBCC, SC, and EC. The rate of overlap of proteins predicted by LBCC and LIDC was 35 percent. The fifth column is the number of true essential proteins in the set *LBCC* − *M*, and the sixth column is the number of true essential proteins in the set *M* − *LBCC*. The number of true essential proteins identified by LBCC was the highest among the prediction methods. In particular, LBCC yielded 50 more true essential proteins than DC, LAC, SC, EC, BC and NC. We also plotted the subgraph of the top 100 proteins predicted by DC and the top 100 proteins predicted by LBCC in Fig 14 and the subgraph of the top 100 proteins predicted by SC and the top 100 proteins predicted by LBCC in Fig 15. The node number of the subgraph is less than 200 if *LBCC* ∩ *DC* ≠ ∅ (or *LBCC* ∩ *SC* ≠ ∅). In the two subgraphs, the blue nodes and green nodes form a dense network, whereas the red nodes and yellow nodes form sparse networks in which there are even several isolated nodes. Hence, the essential proteins identified by LBCC exhibit significant modularity.

**Fig 16:**
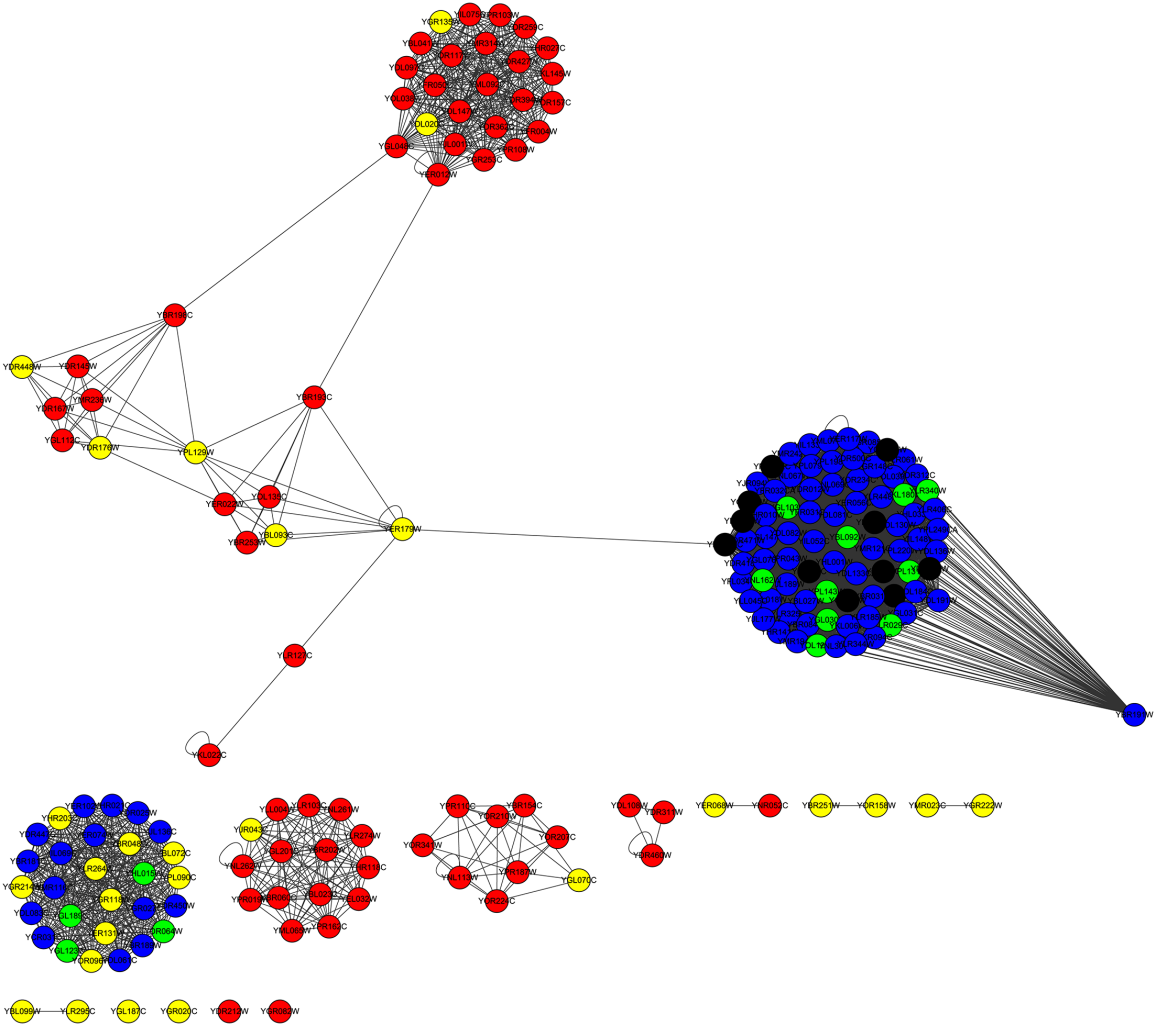
The top 189 proteins in the YMBD network identified by LAC ∪ LBCC. The green nodes and blue nodes are proteins identified by *LAC*; the former are true essential proteins, and the latter are nonessential proteins. The red nodes and yellow nodes are proteins identified by *LBCC*; the former are true essential proteins, and the latter are nonessential proteins. The black nodes are the overlapping proteins.

**Fig 17:**
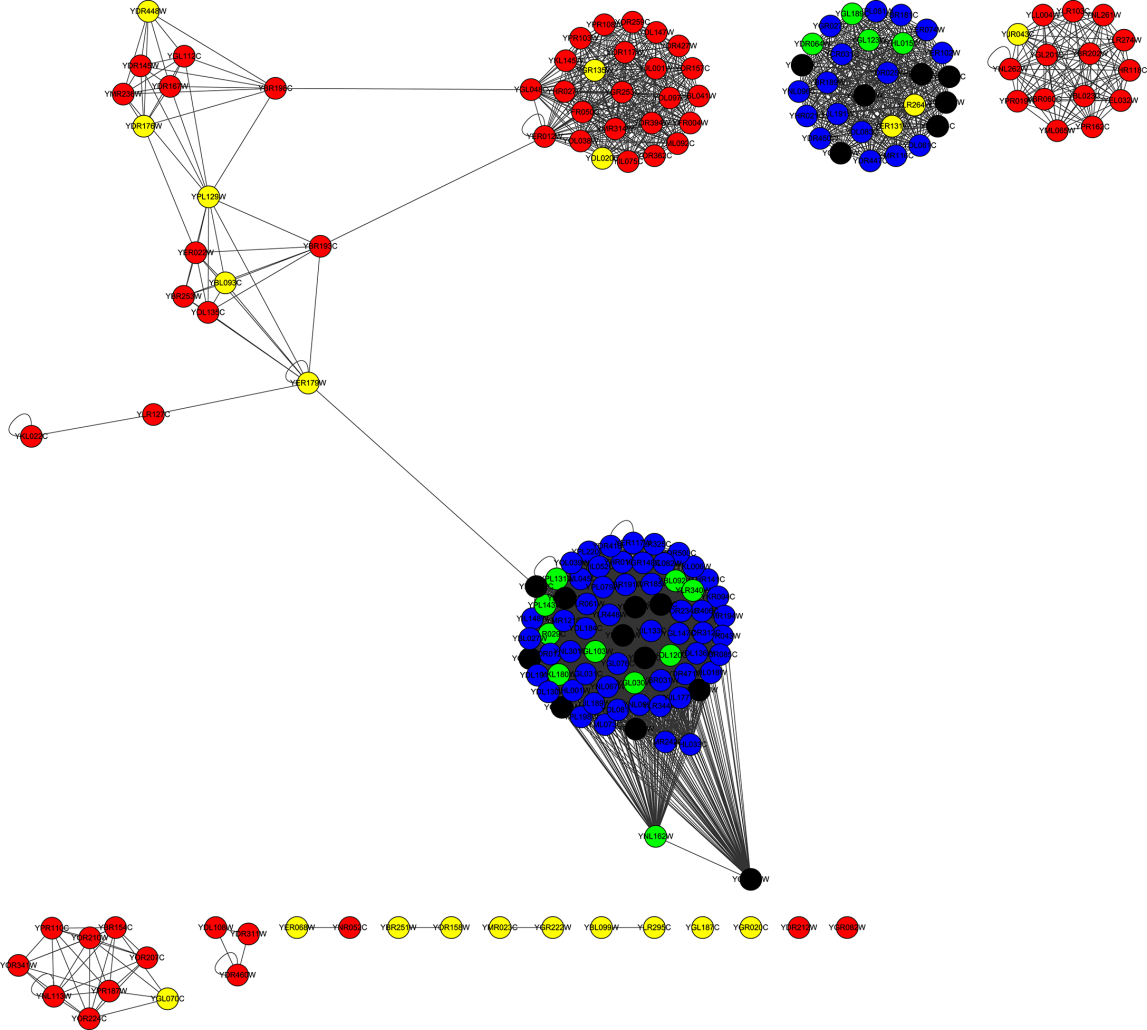
The top 182 proteins in the YMBD network identified by LIDC ∪ LBCC. The green nodes and blue nodes are proteins identified by *LIDC*; the former are true essential proteins, and the latter are nonessential proteins. The red nodes and yellow nodes are proteins identified by *LBCC*; the former are true essential proteins, and the latter are nonessential proteins. The black nodes are the overlapping proteins.

For the YMBD dataset, the column |*LBCC* ∩ *M*| demonstrates that the rate of overlap of proteins predicted by LBCC and the other seven methods was not greater than 20 percent. The fifth and sixth columns show that LBCC predicted 50 more true essential proteins than the other prediction methods, including LIDC. Similarly, we plotted two subgraphs of *LAC* ∪ *LBCC* and *LIDC* ∪ *LBCC*, shown in Figs 16 and 17, respectively. The blue nodes and green nodes form two dense networks, whereas the red nodes and yellow nodes form sparse networks. Hence, the essential proteins identified by LBCC also exhibit stronger modularity.

**Fig 18:**
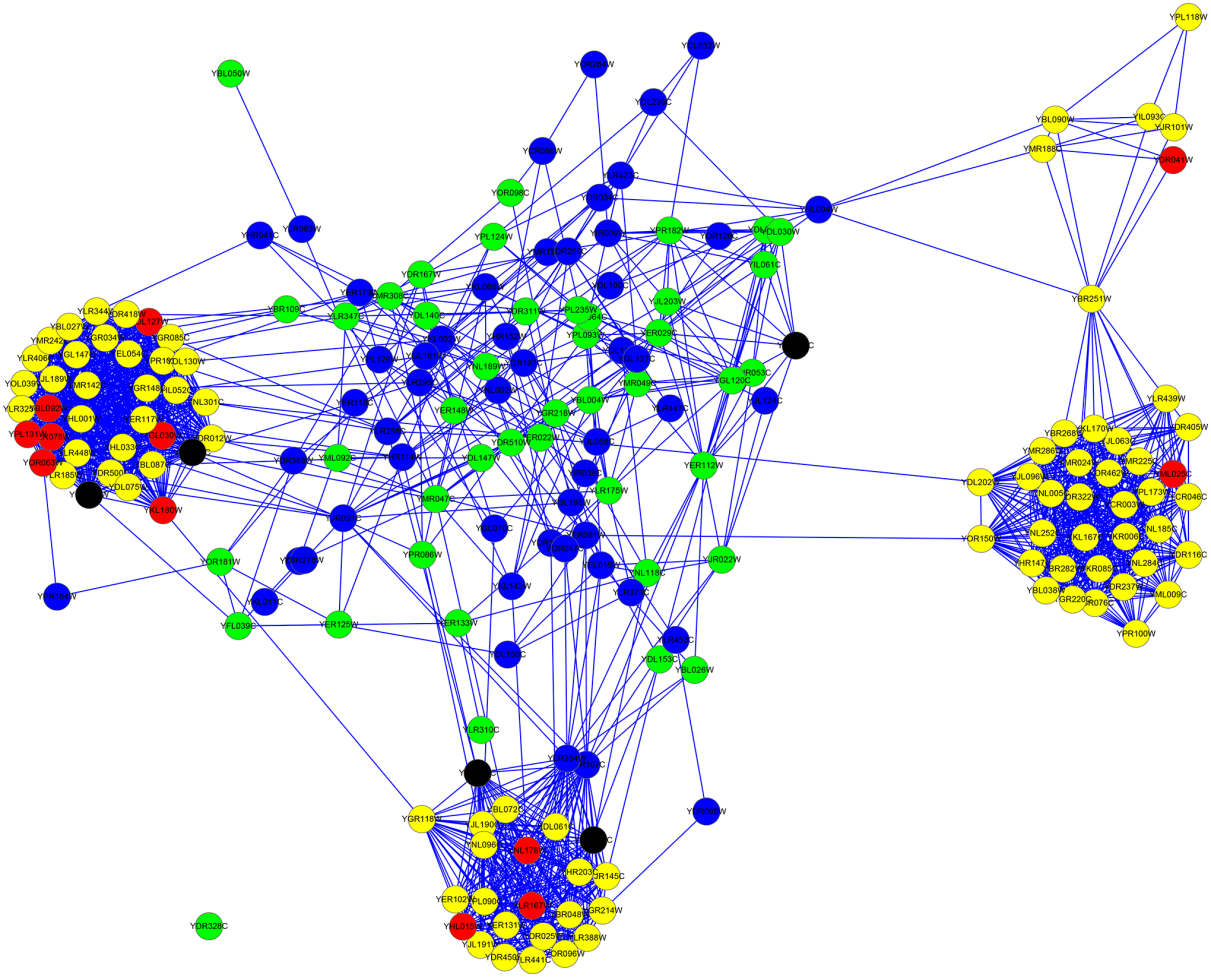
The top 195 proteins in the YHQ network identified by BC ∪ LBCC. The green nodes and blue nodes are proteins identified by *BC*; the former are true essential proteins, and the latter are nonessential proteins. The red nodes and yellow nodes are proteins identified by *LBCC*; the former are true essential proteins, and the latter are nonessential proteins. The black nodes are the overlapping proteins.

**Fig 19:**
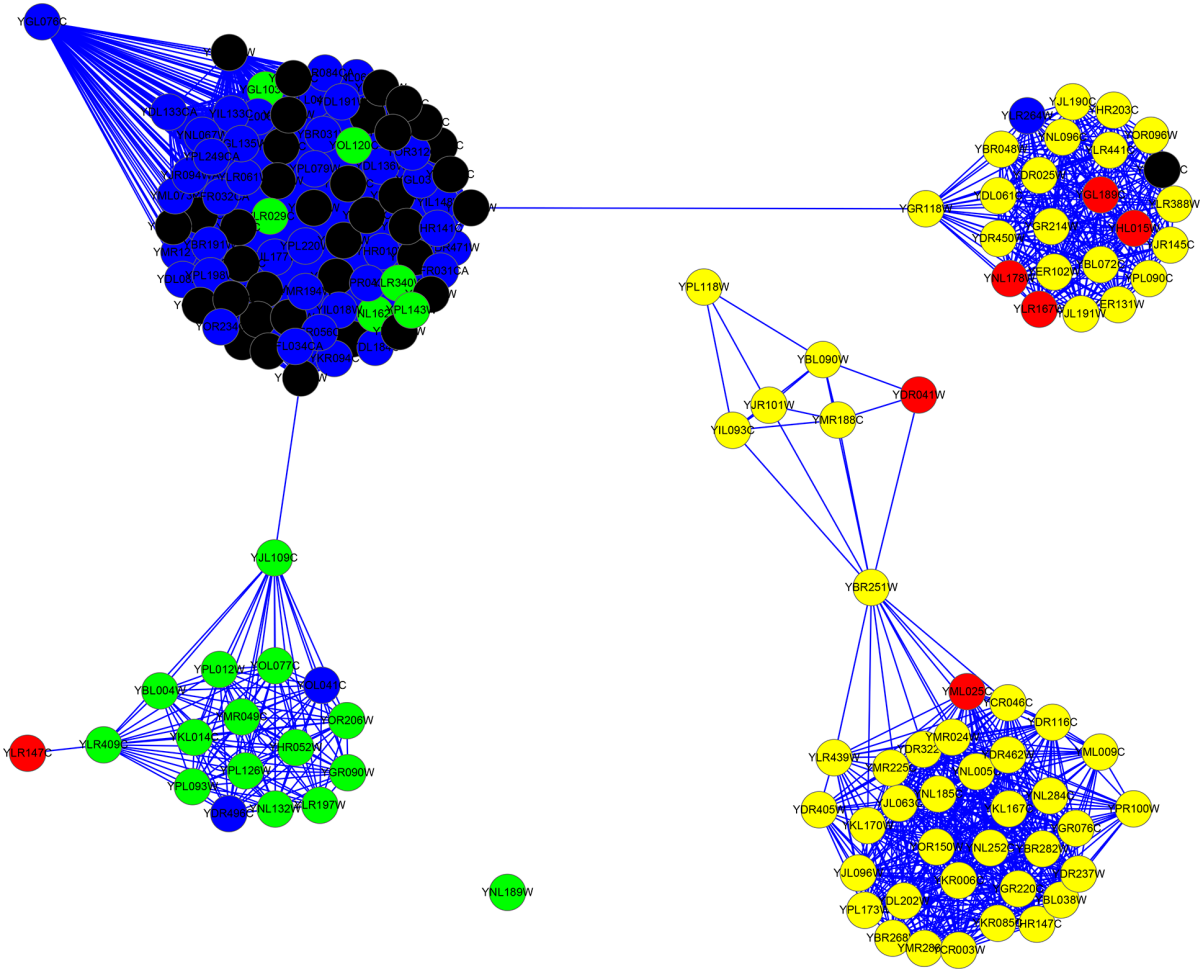
The top 163 proteins in the YHQ network identified by NC ∪ LBCC. The green nodes and blue nodes are proteins identified by *NC*; the former are true essential proteins, and the latter are nonessential proteins. The red nodes and yellow nodes are proteins identified by *LBCC*; the former are true essential proteins, and the latter are nonessential proteins. The black nodes are the overlapping proteins.

For the YHQ dataset, as indicated by column |*LBCC* ∩ *M*|, the rates of overlap of the proteins are less than 40 percent, except for LIDC, for which the rate of overlap is 48 percent. The fifth and sixth columns show that the number of true essential proteins predicted by LBCC is less than those predicted by the other methods due to the less desirable results at the top 100 level (see Fig 4). We also plotted the two subgraphs for *BC* ∪ *LBCC* and *NC* ∪ *LBCC*, shown in Figs 18 and 19, respectively. The blue nodes and green nodes form some dense networks, whereas the red nodes and yellow nodes form four sparse networks. Thus, the essential proteins predicted by LBCC show stronger modularity.

**Fig 20:**
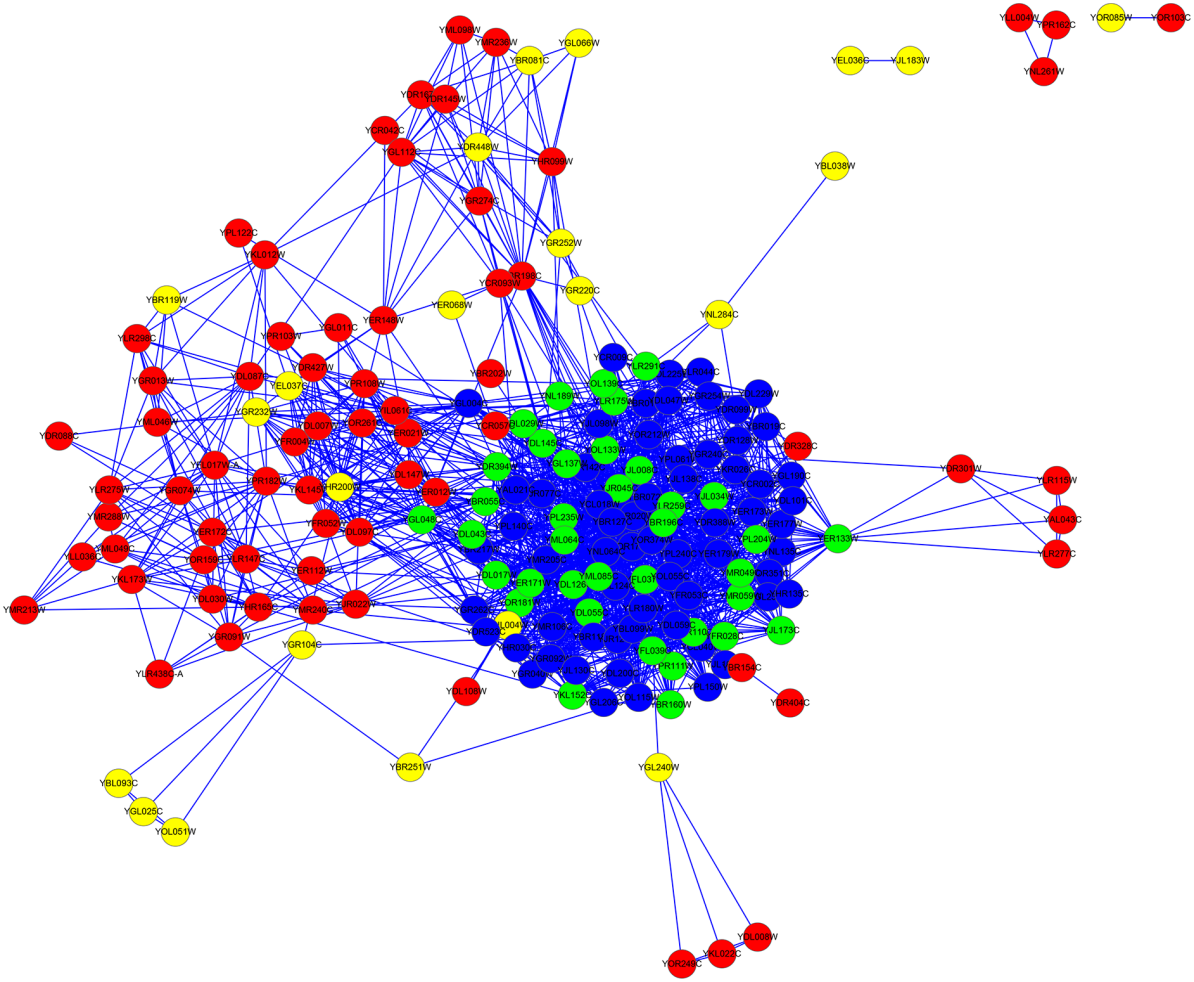
The top 200 proteins in the YDIP network identified by EC ∪ LBCC. The green nodes and blue nodes are proteins identified by *EC*; the former are true essential proteins, and the latter are nonessential proteins. The red nodes and yellow nodes are proteins identified by *LBCC*; the former are true essential proteins, and the latter are nonessential proteins.

**Fig 21:**
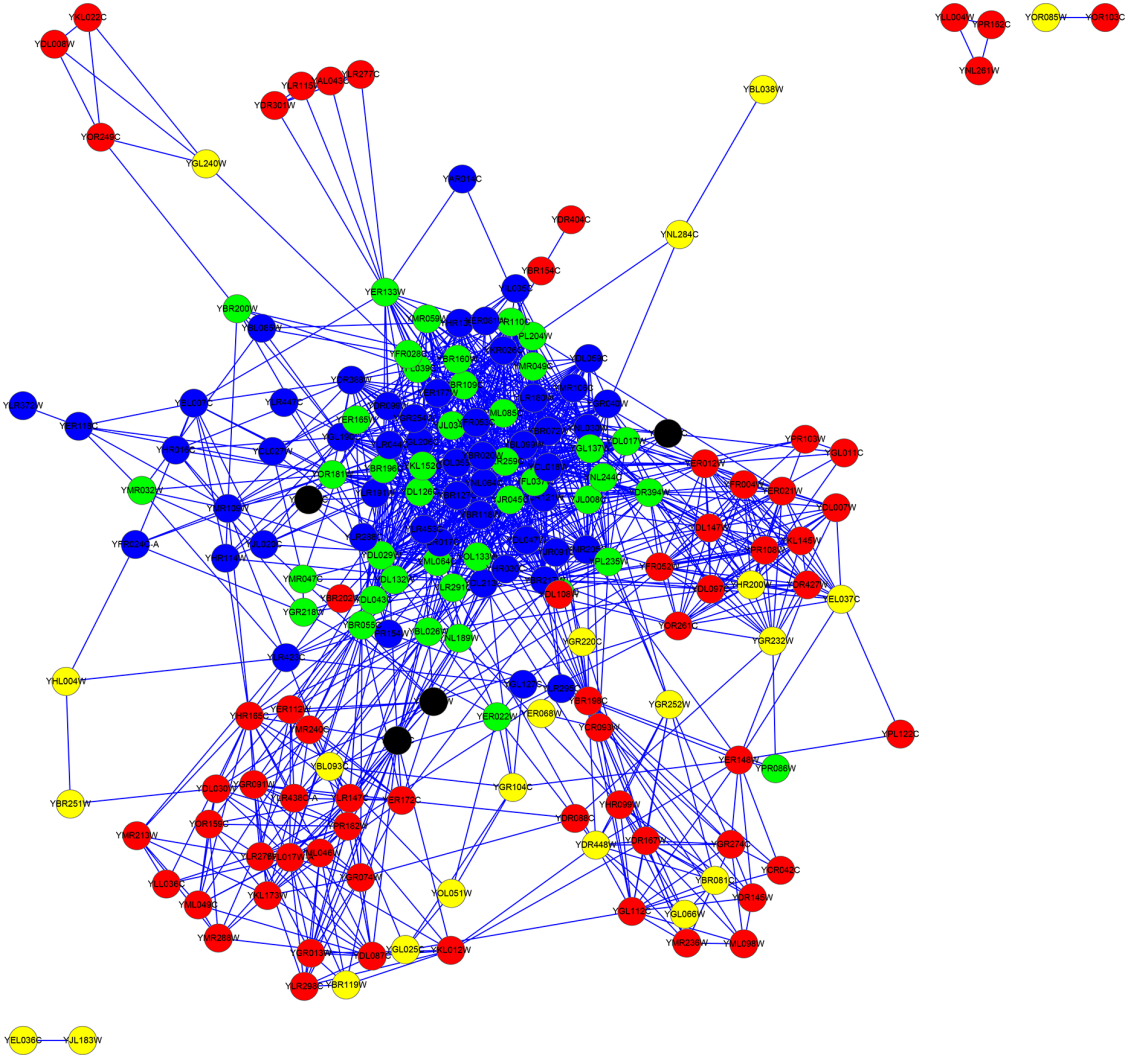
The top 196 proteins in the YDIP network identified by DC ∪ LBCC. The green nodes and blue nodes are proteins identified by *DC*; the former are true essential proteins, and the latter are nonessential proteins. The red nodes and yellow nodes are proteins identified by *LBCC*; the former are true essential proteins, and the latter are nonessential proteins. The black nodes are the overlapping proteins.

For the YDIP dataset, the column |*LBCC* ∩ *M*| shows that the rate of overlap of the proteins predicted by LBCC and the other six methods (DC, LAC, SC, EC, BC, and NC) is less than 30 percent. As indicated by the fifth and sixth columns, the number of true essential proteins predicted by LBCC is greater than those predicted by the other methods (DC, LAC, SC, EC, BC, and NC). Compared with LIDC, the rate of overlap is 66 percent, and 6 fewer true essential proteins are predicted by LBCC compared to LIDC. Similarly, we plotted the two subgraphs for *EC* ∪ *LBCC* and *DC* ∪ *LBCC*, shown in Figs 20 and 21, respectively. The blue nodes and green nodes form dense networks, whereas the red nodes and yellow nodes form some sparse networks. Thus, the essential proteins predicted by LBCC exhibit stronger modularity.

The analysis of the differences between these measures demonstrates that LBCC is significantly different from the other measures and is more accurate in terms of the discovery of essential proteins in most cases.

### Results on human PPI network

To further evaluate the performance of the proposed method LBCC, we also applied it to identify essential proteins on a human PPI network. The human PPI network data marked HDIP were from the DIP database [26], the essential proteins were collected from DEG [29], and the protein complex set marked HCOM was from CORUM (Comprehensive Resource of Mammalian protein complexes) [35]. HDIP consisted of 4647 interactions and 2914 proteins, including 1887 essential proteins, and HCOM contained 1283 protein complexes.

**Fig 22:**
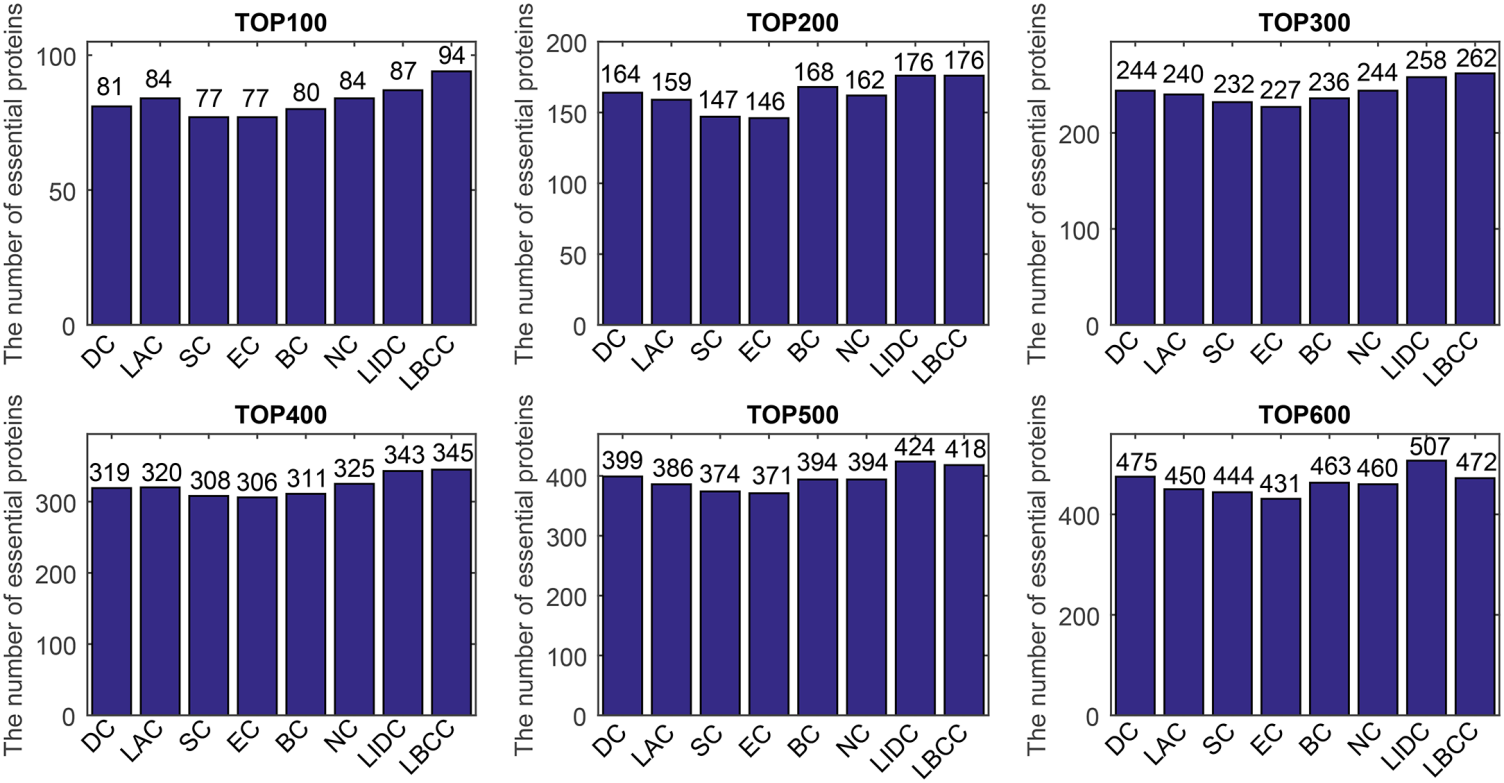
The number of true essential proteins predicted by LBCC and the other seven previously proposed methods for the HDIP network.

First, we compared the performances of LBCC and the other seven methods in six levels from the top 100 to top 600. As shown in Fig 22, almost every method achieved more than 70 percent precision due to the large proportion of essential proteins, and LBCC achieved the best results at the top 100-400 levels. However, LBCC tended to provide less desirable results compared with LIDC at the top 500 and 600 levels.

**Fig 23:**
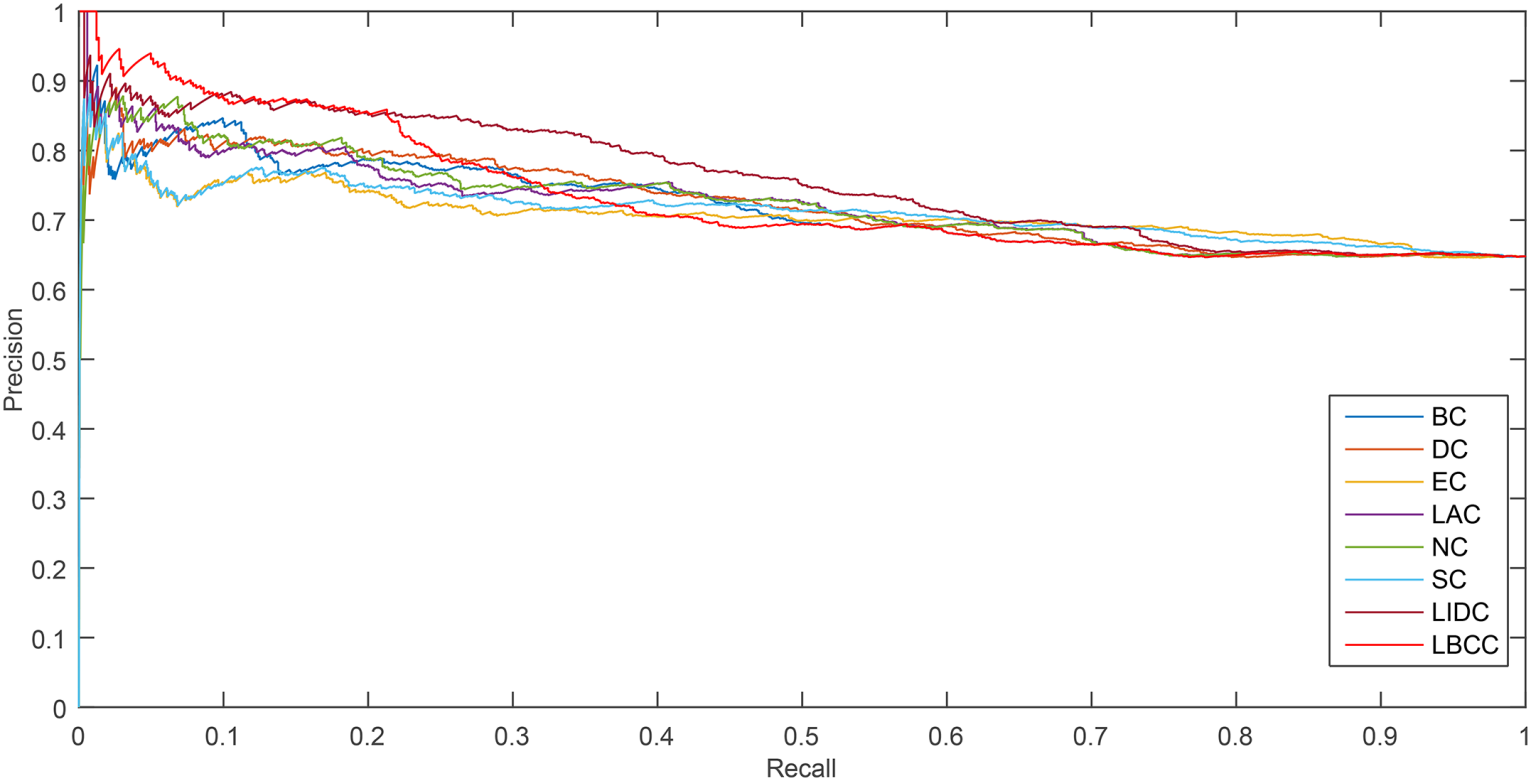
PR curves of LBCC and the other seven previously proposed methods for the HDIP network.

Second, we used six statistical methods and precision-recall curves to evaluate the performance of LBCC and the other methods. As shown in Table 6, the values of the six statistical methods for LBCC were slightly lower than for LIDC. From the precision-recall curves shown in Fig 23, LBCC performed better than the other methods between the recall levels of 0 and 0.22.

**Table 6:** Comparative analysis of LBCC and the other seven previously proposed methods in terms of SN, SP, PPV, NPV, F-measure, and ACC with the HDIP dataset.

Dataset	Methods	SN	SP	PPV	NPV	F-measure	ACC
HDIP	DC	0.244	0.882	0.792	0.389	0.373	0.469
	LAC	0.232	0.860	0.753	0.379	0.355	0.453
	SC	0.230	0.856	0.746	0.377	0.352	0.451
	EC	0.223	0.843	0.723	0.371	0.341	0.442
	BC	0.240	0.873	0.777	0.385	0.366	0.463
	NC	0.235	0.866	0.763	0.381	0.360	0.457
	LIDC	**0.262**	**0.914**	**0.849**	**0.403**	**0.400**	**0.492**
	LBCC	0.245	0.884	0.796	0.389	0.375	0.470

**Fig 24:**
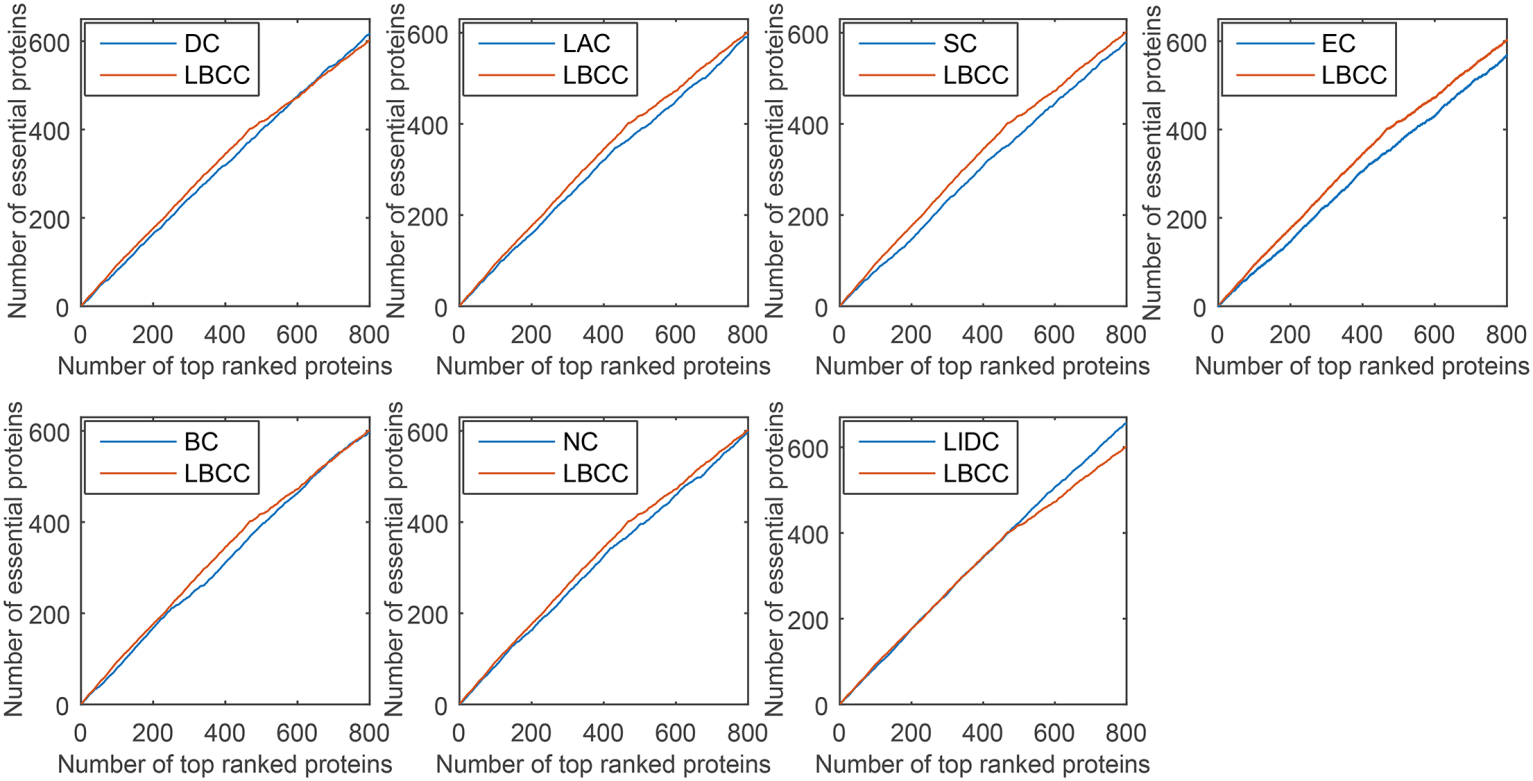
Jackknife curves of LBCC and the other seven previously proposed methods for the HDIP network.

Finally, we used the jackknife methodology to assess the generality of LBCC and the other seven methods. The results are presented in Fig 24, in which LBCC exhibited a performance similar to that of LIDC before the top 500 and superior to LAC, SC, EC and NC. Hence, the LBCC method is also effective for predicting essential proteins for the human PPI network HDIP.

## Conclusion

The identification of essential proteins is helpful for comprehending the minimal requirements for cellular life, and many approaches based on topological properties have been proposed for discovering essential proteins in PPI networks. Most of the topology-based methods only concentrate on either local or global characteristics and are also sensitive to the network structure.

In 2015, Luo and Qi [15] proposed the method LIDC based on information on protein complexes. LIDC outperformed classical topological centrality measures. In this paper, we propose a new method, LBCC, based on the combination of three characteristics of the protein-protein interaction network, i.e., *Den*_1_(*v*), *Den*_2_(*v*), *BC*(*v*) and *IDC*(*v*), which represent both local and global characteristics and information on protein complexes.

We applied LBCC to four PPI networks of *Saccharomyces* *cerevisiae*: YMIPS, YMBD, YHQ and YDIP. We then conducted comprehensive comparisons of LBCC and the other seven previously proposed methods, including DC, BC, SC, EC, NC, LAC and LIDC, in terms of the number of true essential proteins identified. At the six levels from the top 100 to top 600, LBCC outperformed recent prediction methods on the YMIPS and YMBD datasets. In particular, LBCC improved the prediction precision by more than 10 percent compared to LIDC. Based on the analysis of the six statistical methods, precision-recall curve and jackknife methodology for the four datasets, the experimental results demonstrate that LBCC is more stable and general than the recently developed prediction methods in most cases. Moreover, we also applied LBCC to a human PPI network, HDIP. The experimental results show that LBCC is also effective for predicting essential proteins for the HDIP network.

Hence, we conclude that LBCC is a more effective method for predicting essential proteins, occasionally significantly. In future studies, we will integrate additional information, such as domain information, gene ontology and gene expression data, to predict essential proteins more effectively and accurately.

## Supporting Information

S1 Excel Essential protein and nonessential protein data. (XLS)

S2 Excel Protein complex data. (XLSX)

S1 Text Protein interaction data in YMIPS. (TXT)

S2 Text Protein interaction data in YMBD. (TXT)

S3 Text Protein interaction data in YHQ. (TXT)

S4 Text Protein interaction data in YDIP. (TXT)
